# Aggregation shifts amyloid-**β** peptides from synaptogenic to synaptotoxic

**DOI:** 10.1172/JCI193407

**Published:** 2025-09-30

**Authors:** Alberto Siddu, Silvia Natale, Connie H. Wong, Hamidreza Shaye, Thomas C. Südhof

**Affiliations:** 1 Deptartment of Molecular and Cellular Physiology and; 2 Howard Hughes Medical Institute, Stanford University School of Medicine, Stanford, California, USA.

**Keywords:** Cell biology, Neuroscience, Alzheimer disease, Molecular biology, Synapses

## Abstract

Whether amyloid-β (Aβ) peptides are synaptogenic or synaptotoxic remains a pivotal open question in Alzheimer’s disease research. Here, we chronically treated human neurons with precisely controlled concentrations of chemically defined synthetic Aβ40, Aβ42, and Aβ42^arctic^ peptides that exhibit distinct aggregation propensities. Remarkably, chronic exposure of human neurons to free Aβ40 at higher concentrations or to free Aβ42 at lower concentrations potently promoted synapse formation. In contrast, aggregated Aβ42 or Aβ42^arctic^ at higher concentrations were neurotoxic and synaptotoxic. The synaptotoxic effects of Aβ peptides manifested as an initial contraction of the synaptic vesicle cluster followed by synapse loss. Aβ40 and Aβ42 peptides with scrambled or inverted sequences were inactive. Thus, our experiments reveal that Aβ peptides exhibit an aggregation-dependent functional dichotomy that renders them either synaptogenic or synaptotoxic, thereby providing insight into how Aβ peptides straddle a thin line between physiological synapse organization and pathological synapse disruption. Among others, our data suggest that Alzheimer’s disease therapies might aim to shift the balance of Aβ peptides from the aggregated to the free state instead of suppressing all Aβ peptides.

## Abstract 2

Free Aβ peptides promote synapse formation in human neurons, whereas aggregated forms are synaptotoxic, highlighting a delicate balance critical for Alzheimer’s disease therapeutic strategies.

## Introduction

Alzheimer’s disease (AD) is characterized by a relentless decline in cognitive function, underpinned by progressive synapse loss and neuronal cell death. Central to the pathology of AD are 2 hallmarks, amyloid plaques and neurofibrillary tangles, often accompanied by widespread neuroinflammation (1). Although multiple pathogenic processes likely contribute to AD pathogenesis, amyloid plaques that are composed predominantly of aggregated amyloid-β (Aβ) peptides are considered central drivers of AD pathogenesis. According to the “amyloid hypothesis” that was conceived in 1991 by Selkoe and Hardy, the toxicity of various forms of Aβ peptides causes AD, leading to a vast effort to suppress the production or the concentration of Aβ peptides in AD patients as a therapeutic strategy (2).

Two major lines of evidence provide compelling support for the amyloid hypothesis. First, point mutations in genes encoding the amyloid precursor protein (APP) — the precursor of Aβ peptides and, in presenilins, the catalytic subunits of the intramembrane protease that produce Aβ peptides — cause familial AD with nearly 100% penetrance. These mutations appear to drive AD pathogenesis by increasing the production of Aβ peptides or facilitating their aggregation (3). This genetic link has placed Aβ squarely at the center of AD research, highlighting aggregated Aβ as a principal suspect in initiating or at least accelerating the synaptic dysfunction that is an early event in AD pathogenesis. Second, clinical trials harnessing antibodies against aggregating Aβ demonstrated that limiting Aβ aggregation provides a significant, albeit modest, benefit to AD patients by decreasing the rate of cognitive decline (4–6). These trials offered a crucial proof-of-concept for the notion that Aβ peptide aggregation is indeed at least contributing to AD pathogenesis.

However, multiple observations also indicate that the amyloid hypothesis by itself does not explain AD pathogenesis. In familial AD, it takes 4–5 decades for patients to develop cognitive symptoms, suggesting that simply producing more toxic Aβ peptides and their aggregates does not directly cause AD. In clinical trials showing a benefit of Aβ peptide antibodies in AD, cognitive decline continued even after Aβ plaques were largely cleared. Moreover, in these trials the free Aβ peptide concentrations increased in parallel with the therapeutic benefits. Historically, the antibodies that preferentially bind to aggregated Aβ showed the most power in ameliorating AD symptoms, whereas antibodies that also targeted free Aβ peptides did not produce a significant benefit (7–9). Furthermore, many genetic traits predisposing individuals to AD affect genes encoding microglial proteins, suggesting that the robust inflammatory response to AD contributes to AD pathogenesis by an unknown mechanism (10).

These results motivated the field of AD research to adopt a more nuanced view of the role of Aβ peptides in AD pathogenesis, a role in which Aβ peptides might exert more than a toxic effect. Consistent with this notion, experimental models ranging from cultured neurons to in vivo systems have revealed that aggregated but not free Aβ peptides are toxic (11–17). Our own earlier studies, however, demonstrated that increasing Aβ42 production in human neurons via a conditional APP^Swedish^ knockin mutation was neither synaptotoxic nor neurotoxic (18). Instead, the genetic elevation of Aβ42 production unexpectedly enhanced synapse numbers and bolstered synaptic connectivity, an observation that runs counter to the classic notion that more Aβ automatically equates to greater neurotoxicity. Together, these clinical and experimental findings raise the possibility that free Aβ could fulfill an essential, even beneficial, physiological role at synapses distinct from the role of aggregated Aβ peptides that are neurotoxic, and that this role might become disrupted when the balance shifts toward aggregation.

To investigate the complex interplay between free and aggregated Aβ, here, we systematically examined the impact of chemically defined synthetic Aβ peptides on human neurons under rigorous and precisely controlled conditions. By choosing synthetic peptides, we sought to eliminate confounding variables associated with cellular secretion and processing events, thereby allowing us to isolate and test the direct effects of distinct Aβ species (18). We focused on 3 variants of Aβ peptides, Aβ40, Aβ42, and Aβ42^arctic^, which carries the pathogenic “arctic” mutation (E693G) (referred to as Aβ42^arctic^) (19). We selected Aβ40 due to its relatively slow aggregation kinetics, Aβ42 because of its propensity to aggregate more rapidly, and Aβ42^arctic^ for its “super-aggregating” capacity. By spanning this range of aggregation tendencies, we aimed to capture the spectrum of Aβ effects relevant to both physiological and pathological states. Moreover, we used peptides with a scrambled and a reverse sequence to control for nonspecific physicochemical effects. Our results reveal that Aβ peptides possess a remarkable functional duality: under conditions that minimize aggregation, Aβ peptides are synaptogenic and facilitate normal synaptic communications. However, once Aβ peptides aggregate, these very same Aβ peptide species induce profound synapto- and neurotoxicity.

Our findings thus suggest that the pathogenic potential of Aβ rests heavily on its aggregation state, shifting it from a physiological modulator to a dangerous neurotoxin. Because our experiments used chemically pure synthetic peptides, free from the complexities of cellular expression and secretion, these findings identify Aβ peptides as agents that directly mediate both synaptogenicity and synaptotoxicity. We therefore suggest that AD progression could be driven both by the gain of synaptotoxicity and the loss of synaptogenicity. In our experiments, Aβ peptides are synaptotoxic before they are neurotoxic and produce a distinctive pattern of changes in synapse morphology, indicative of a selective impairment of presynaptic vesicle dynamics. The discovery of the functional dichotomy of Aβ peptides illuminates the nuanced landscape of AD pathogenesis, suggesting that therapies might aim to not only suppress deleterious Aβ peptide aggregates but also to support the functions of free Aβ peptides.

## Results

### Aβ toxicity.

**Figure 1:**
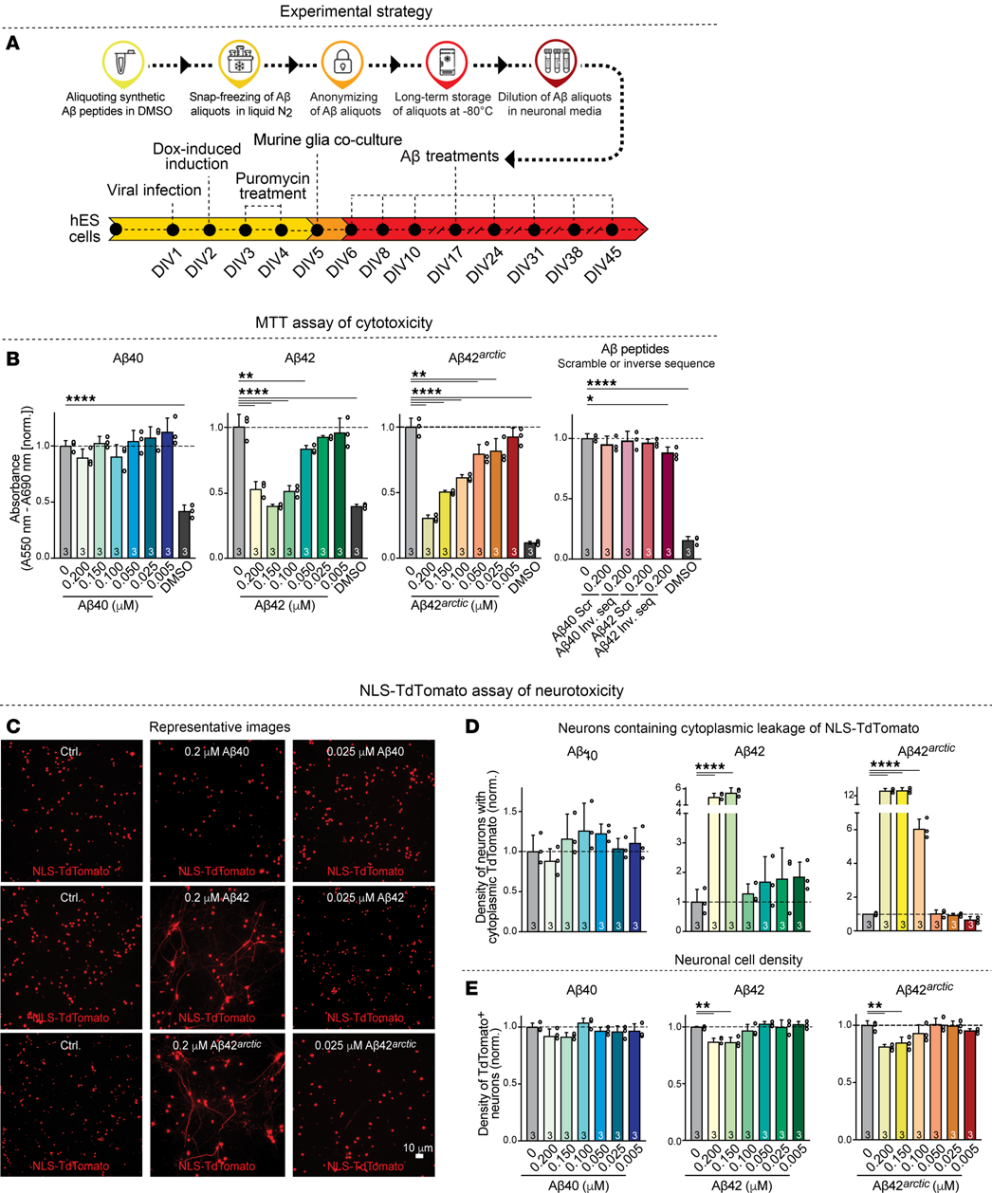
Chemically defined Aβ42 and Aβ42^arctic^ peptides, but not Aβ40 peptides, display robust toxicity in human neurons. (**A**) Experimental strategy. The experimental design results in chronic exposure of human neurons to Aβ peptides at defined concentrations for 39 days. Note that the Aβ peptides were not exposed to aggregating conditions prior to culture additions. Once added, however, at least Aβ42 and Aβ42^arctic^ are likely subject to dynamic aggregation. As controls, Aβ40 and Aβ42 peptides with scrambled or reverse sequences were used. (**B**) Measurements of the toxicity of various Aβ peptides at the indicated concentrations using the MTT assay for cell damage (21). Treatments with DMSO (10%) were included as a highly toxic positive control. The effects of scrambled or reverse-sequence control peptides are shown on the right. (**C** and **D**) Measurements of the toxicity of Aβ peptides at the indicated concentrations using the NLS-TdTomato assay that scores the leakage of NLS-TdTomato containing a nuclear localization signal from the nucleus into the cytoplasm. NLS-TdTomato only leaks into the cytoplasm when a cell’s energy state is compromised and ATP levels decline. (**C**) Representative images; scale bar: 10 μm. (**D**) Summary graphs of the density of neurons containing cytoplasmic NLS-TdTomato). (**E**) Quantification of the neuronal cell density measured as the density of NLS-TdTomato-positive nuclei to demonstrate that only high concentrations of Aβ42 and Aβ42^arctic^ cause significant cell death that is modest at the relatively low Aβ42 and Aβ42^arctic^ concentrations used. All neurons were analyzed at DIV45. All numerical data are means ± SEM; numbers of independent experiments are reported in the bars (*n* = 3). Statistical significance was assessed as prespecified by 1-way ANOVA with post hoc corrections, comparing the mean of each group with control (Ctrl), with **P < 0.01, and ****P < 0.0001. Nonsignificant comparisons are not indicated. Additional representative images, analyses of the data as pseudo replicates, validation of the NLS-TdTomato assay, and confirmation of the results with a third cell toxicity assay in addition to the MTT assay and the NLS-TdTomato assay are shown in Supplemental Figure 1. *P < 0.05; **P < 0.01; ***P <.001; ****P <.0001.

To ensure reproducible applications of Aβ peptides, we reconstituted lyophilized synthetic Aβ peptides in DMSO at 1 mg/mL before the start of an experiment. The identity of the Aβ peptides was validated by mass spectrometry (see Supplemental Data File 1; supplemental material available online with this article; https://doi.org/10.1172/JCI193407DS1). We then generated a dilution series of the reconstituted peptides in DMSO, snap froze aliquots of each dilution in liquid nitrogen, and stored the aliquots at –80°C. To mimic chronic Aβ exposure conditions, we treated human neurons with Aβ peptides at concentrations ranging from 0.005 to 0.2 μM by adding them during each media change, regularly performed from days in vitro (DIV)6 to DIV45 (Figure 1A). For each Aβ peptide-addition time point, we thawed a fresh vial for a given Aβ peptide concentration and added the Aβ peptide to the human neuron culture medium; no peptides were frozen twice. Treatments were coded to avoid observer bias. With this experimental design, all peptides are present in our experiments in an aqueous solution for a precisely defined time to control for the time-dependent aggregation of Aβ peptides that is fastest for Aβ42^arctic^ and slowest for Aβ40 (19, 20).

We first evaluated the neurotoxicity of the 3 Aβ peptides (Aβ40, Aβ42, and Aβ42^arctic^) at various concentrations using 3 independent assays. The MTT assay employs yellow tetrazolium dye that is reduced to a purple formazan crystal by viable cells with active metabolism (21). Because it measures metabolic activity in all cells, this assay serves as a broad cytotoxicity read out for the entire glia/neuron coculture system. The amount of formazan produced is then measured because it is directly proportional to the number of viable cells (21) (Figure 1B and Supplemental Figure 1A). The nuclear localization TdTomato assay, conversely, enables monitoring of cellular stress by tracking the localization of fluorescent TdTomato fused to a nuclear localization signal. Under healthy conditions, the nuclear localization signal directs the NLS-TdTomato to the nucleus. When toxicity in a cell causes a reduction in ATP levels, NLS-TdTomato leaks out of the nucleus into the cytoplasm, which we scored as an index of toxicity (Figure 1, C and D, and Supplemental Figure 1, B–D). Because the reporter is expressed only in neurons, this assay specifically assesses neurotoxicity within the coculture. We developed this assay because it can be performed on live cells and reflects decreases in ATP levels in these cells. The NLS-TdTomato assay was validated by treating cultures with 5% DMSO and 200 μM hydrogen peroxide, 2 well-established neurotoxins. Both treatments rapidly induced the nuclear-to-cytoplasmic shift of NLS-TdTomato, confirming the assay’s sensitivity and reliability in detecting toxic stress (Supplemental Figure 1B). The Incucyte Cytotox Green assay, finally, monitors membrane integrity using a DNA dye that gains access to nuclei only when the plasma membrane is compromised (Supplemental Figure 1, F and G).

At DIV45, when human neurons are relatively mature, chronic exposure of human neurons to Aβ40 even at high concentrations did not produce any neurotoxicity as measured with either assay (Figure 1, B–D, and Supplemental Figure 1, A, C, and D). In contrast, Aβ42 and Aβ42^arctic^ were neurotoxic at concentrations of greater than or equal to 0.05 μM (Aβ42) or greater than or equal to 0.025 μM (Aβ42^arctic^, as monitored by the MTT assay (Figure 1B and Supplemental Figure 1A). Moreover, Aβ42 and Aβ42^arctic^ were also neurotoxic when analyzed by the NLS-TdTomato assay. With this assay, neurotoxicity became apparent at Aβ peptide concentrations of greater than or equal to 0.15 μM (Aβ42) or of greater than or equal to 0.1 μM (Aβ42^arctic^), possibly because the NLS-TdTomato assay may be less sensitive than the MTT assay (Figure 1, C and D, and Supplemental Figure 1, C and D). Notably, the toxicity of the Aβ42^arctic^ peptide is more pronounced than that of WT Aβ42 at the same concentrations, suggesting that the arctic mutation — which increases Aβ aggregation — also enhances toxicity as described previously (11, 19) (Figure 1, B–D, and Supplemental Figure 1, A, C, and D). The Incucyte Cytotox Green assay gave essentially the same results as the NLS-TdTomato assay. Here, again, neurotoxic doses of Aβ42 and Aβ42^arctic^ produced a modest but consistent rise in labeled nuclei that matched the changes observed with the NLS-TdTomato assay, whereas Aβ40 had no effect, as in the other assays (Supplemental Figure 1, F and G).

To verify that the neurotoxic effects of Aβ42 and Aβ42^arctic^ are sequence specific, we repeated the MTT neurotoxicity analyses with mutant Aβ40 and Aβ42 peptides containing scrambled or inverted sequences. These control peptides behaved statistically indistinguishably from vehicle at every dose. They induced no decrease in formazan production in the MTT assay and therefore exhibited no detectable toxicity. Thus, the physicochemical properties of Aβ42 and Aβ42^arctic^ peptides that include both charged and hydrophobic residues cannot explain their neurotoxicity, which appears to depend on their precise amino acid sequences and not on their amino acid composition (Figure 1B and Supplemental Figure 1A). None of the Aβ treatments caused a large loss of neurons, although higher concentrations of Aβ42 or Aβ42^arctic^ did decrease neuronal cell numbers (approximately 15%). Together, these data suggest that higher concentrations of Aβ42 and Aβ42^arctic^ impair the viability of human neurons and subsequently induce neuronal cell death (Figure 1E and Supplemental Figure 1E). Note that, in these experiments, even the higher doses of Aβ42 and Aβ42^arctic^ we applied were relatively lower than those generally used in the literature, possibly accounting for the lower degree of neurotoxicity we detected.

**Figure 2:**
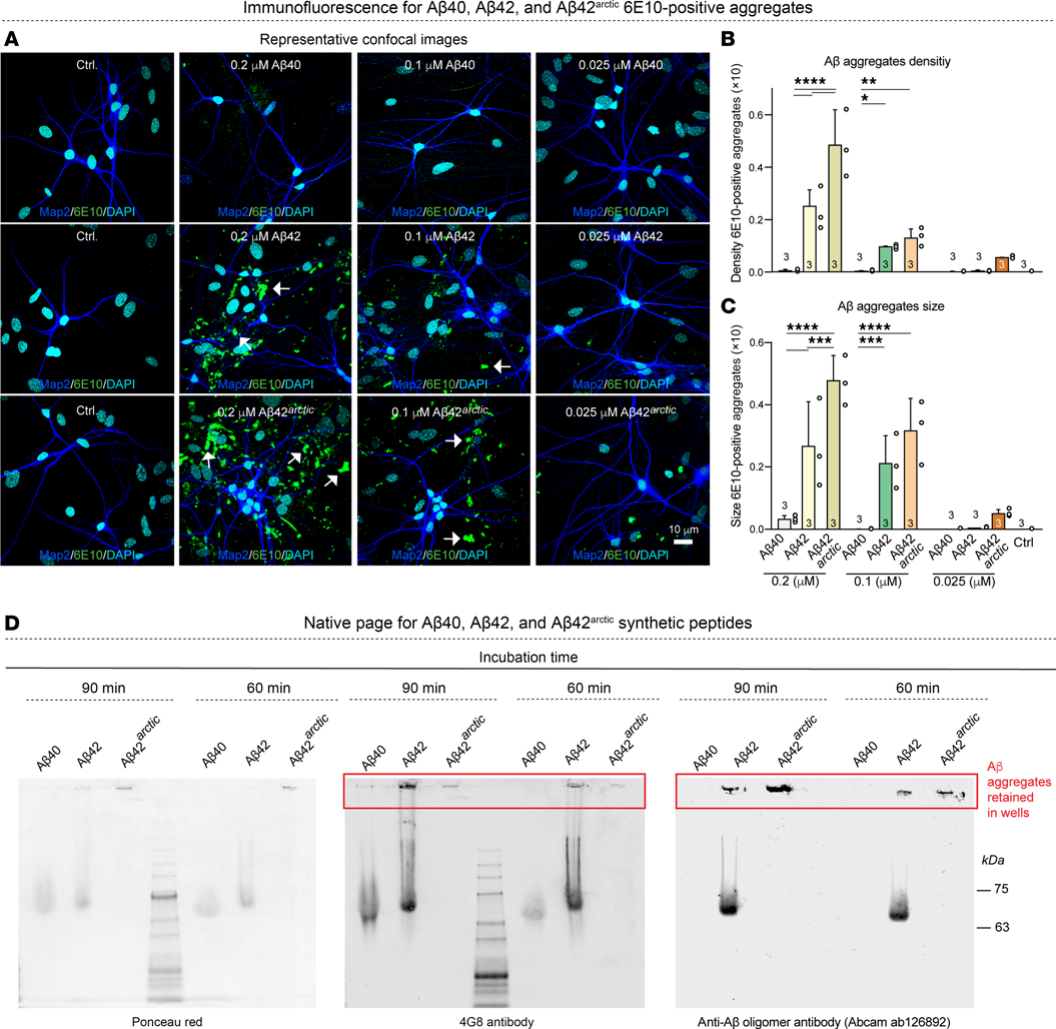
The neurotoxicity of Aβ42 and Aβ42^arctic^ correlates with their aggregation state, whereas Aβ40 under our conditions of use does not exhibit any aggregation. (**A**–**C**) Analyses of the density and size of 6E10-positive aggregates observed in human neurons cocultured with mouse glia (**A**) representative images of human neurons immunolabeled for the dendritic marker MAP2 (blue), Aβ marker 6E10 (green), and additionally stained with the nuclear marker DAPI (cyan); arrows indicate 6E10 positive aggregates. Scale bar: 10 μm. (**B**) Summary graphs of the 6E10-positive aggregates density measured as the number of structures per area of field of view as a function of the indicated conditions. (**C**) Summary graphs of the sizes of 6E10-positive aggregates calculated as area fraction occupied by aggregates in the total area of the field of view. (**D**) Image of immunoblots obtained after native polyacrylamide gel electrophoresis of synthetic Aβ40, Aβ42, and Aβ42^arctic^ peptides after incubation at 37**°**C for 60 or 90 minutes. Blots were stained with Ponceau red or immunostained with the general Aβ antibody 4G8 or with an antibody that preferentially reacts with Aβ aggregates (Abcam 126892). Note that Aβ42^arctic^ peptides are quantitatively aggregated and thus do not enter the gel but are retained in the wells (red boxes). All neurons were analyzed at DIV25. All numerical data are mean ± SEM; numbers of independent experiments are reported in the bars (*n* = 3). Statistical significance was assessed as prespecified by 1-way ANOVA with post hoc corrections, comparing the mean of each group with control (Ctrl). *P < 0.05; **P < 0.01; ***P <.001; ****P <.0001. Nonsignificant comparisons are not indicated. Additional representative images and analyses of the data as pseudo replicates as well as additional toxicity data are shown in Supplemental Figure 2.

To corroborate the link between Aβ aggregation and Aβ toxicity, we visualized Aβ aggregates in treated human neurons using immunofluorescence labeling with the 6E10 antibody (Figure 2, A–C, and Supplemental Figure 2, A and B). We detected almost no aggregates upon chronic exposure of neurons to Aβ40, mirroring its minimal toxicity profile. In contrast, Aβ42- and especially Aβ42^arctic^-treated coverslips contained significant 6E10-positive Aβ aggregates at concentrations correlating with the onset of toxicity. These aggregates were evident in both human neurons (Figure 2, A–C, and Supplemental Figure 2, A and B) and murine primary neuronal cultures analyzed as an additional confirmation (Supplemental Figure 2, C–F), reinforcing the conclusion that the formation of 6E10-positive aggregates coincides with the shift to neurotoxic outcomes. Conversely, at lower, nonaggregating Aβ concentrations —where Aβ peptides exhibit synaptogenic properties— 6E10-positive aggregates were sparse or absent. Moreover, we performed native gel analyses that confirmed that under our conditions, Aβ40 peptides were not aggregated even at high concentrations, whereas Aβ42^arctic^ was completely aggregated even at lower concentrations (Figure 2D). To examine more directly which assemblies drive toxicity, we preaggregated Aβ42 at 37 °C, centrifuged the suspension, and separated a supernatant enriched in monomers and small oligomers from a pellet containing large aggregates and high molecular weight oligomers. Native PAGE and immunoblotting validated this fractionation (Supplemental Figure 2, G–I). When each fraction was applied chronically to neurons and evaluated by the MTT assay at DIV45, both induced the same loss of viability. Because the pellet consists of aggregated species while the supernatant retains a mixture of small and residual large assemblies, these results implicate aggregated Aβ42 as the principal toxic entity, although a minor contribution from smaller species cannot be excluded.

**Figure 3:**
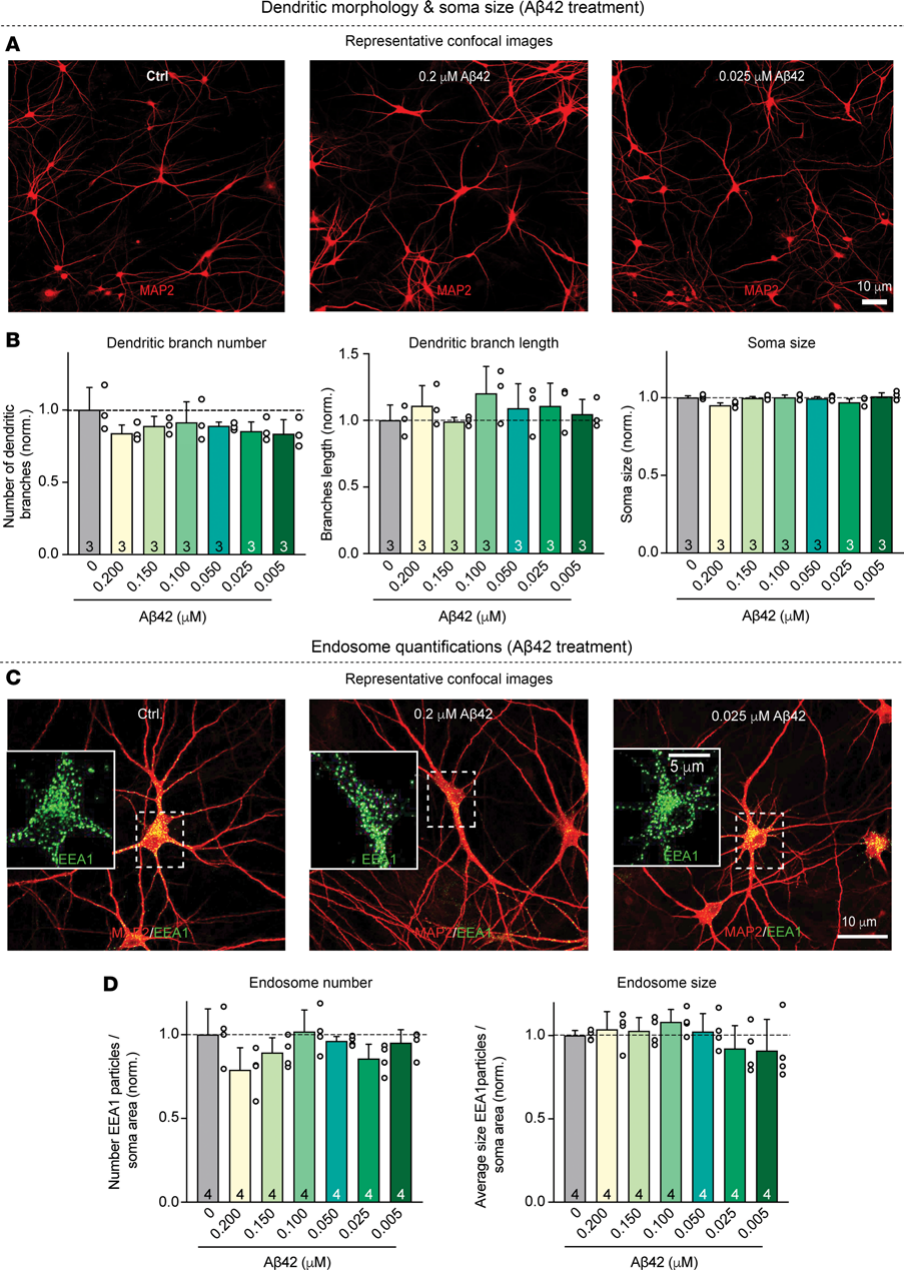
Chronic treatment of human neurons with various concentrations of Aβ42 does not significantly alter their dendritic arborization, soma size, endosome numbers, or endosome size. (**A** and **B**)Analysis of the dendritic arborization and soma size of neurons as a function of the chronic treatment with Aβ42 fails to reveal major changes (**A**) representative images of neurons immunolabeled for the somato-dendritic marker MAP2; (**B**) summary graphs of the dendritic branch numbers and lengths and the soma size). (**C** and **D**) Analysis of endosomes in neurons as a function of the chronic treatment with Aβ42 does not uncover significant changes produced by any concentration of Aβ42. (**C**) Representative images of neurons stained for MAP2 and the endosomal marker EEA1 (insets, expanded single-channel EEA1 images of the neuronal soma). (**D**) Summary graphs of density and sizes of the EEA1-positive somatic endosomes as a function of Aβ42 concentration. Human neurons were chronically treated with the indicated concentrations of synthetic Aβ42 peptides as described in Figure 1A and examined by confocal microscopy at DIV45. All numerical data are mean ± SEM; numbers of true replicates are reported in the bars (*n* = 3–4). Statistical significance as prespecified was assessed by 1-way ANOVA with post-hoc corrections, comparing the mean of each group with control (Ctrl). Nonsignificant comparisons are not indicated. More representative images and analyses of the data as pseudo replicates are shown in Supplemental Figure 3. Scale bars: 10 μm; 5 μm (inset).

Taken together, these experiments confirm previous conclusions (11–19) that Aβ40, Aβ42, and Aβ42^arctic^ follow distinct aggregation trajectories and demonstrate that their aggregation directly predicts their neurotoxicity. We then asked whether chronic treatments of human neurons with Aβ42 at nonneurotoxic and neurotoxic concentrations produce changes in dendritic arborization, soma size, or endosomes in human neurons. Remarkably, we detected no changes in any of these parameters even at toxic Aβ42 concentrations (Figure 3, A–D and Supplemental Figure 3, A–D). Thus, the prolonged chronic treatment of human neurons with Aβ42 peptides does not simply kill neurons or impair the cellular organization but induces a progressive impairment of the neurons that eventually leads to cell death.

### Aβ synaptogenicity.

**Figure 4:**
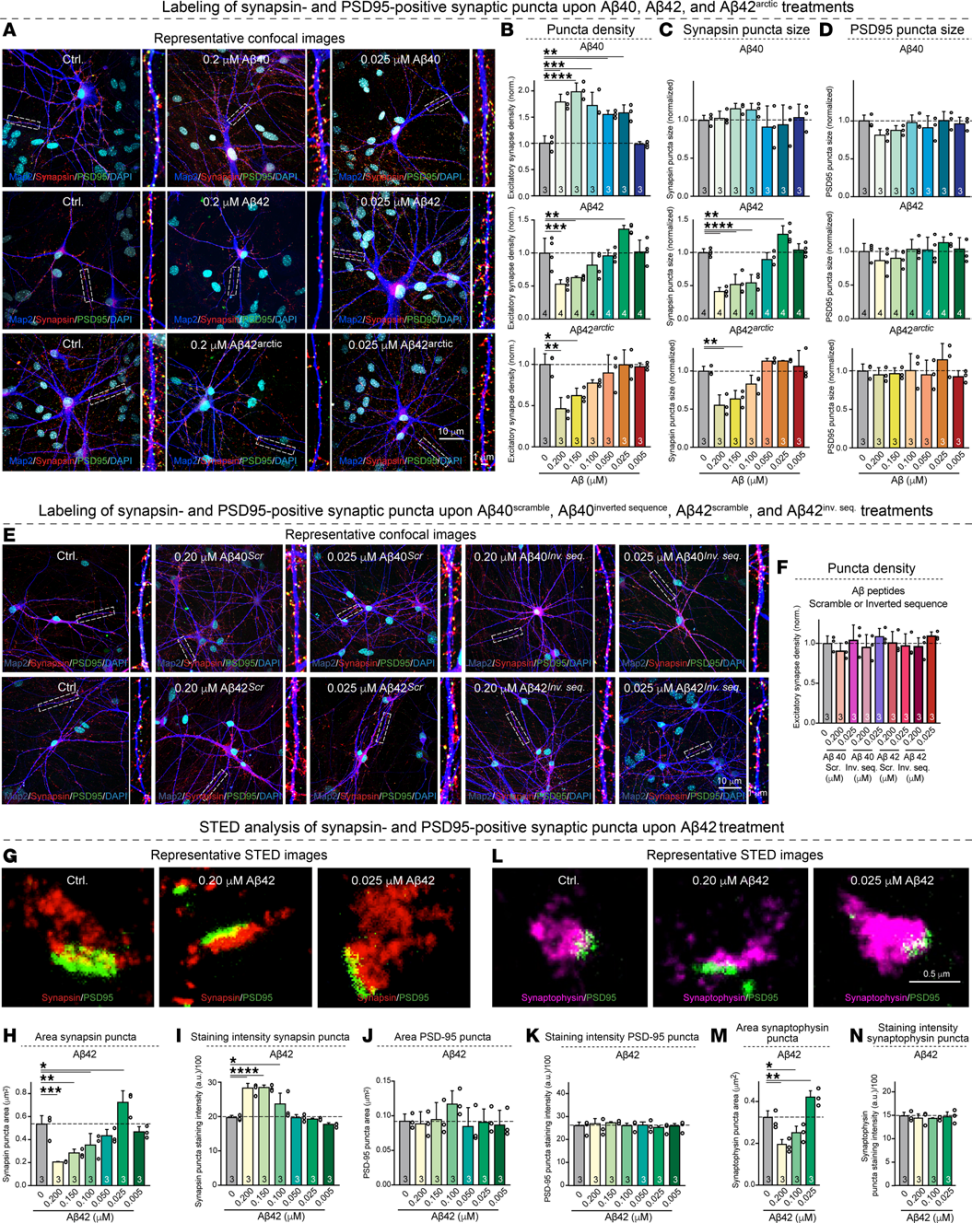
Analyses of the synaptogenic and synaptotoxic actions of Aβ40, Aβ42, and Aβ42^arctic^ peptides and of the scrambled and inverted sequence versions of Aβ40 and Aβ42 in human neurons by quantitative imaging. (**A**–**D**) Analyses of the density and size of synapsin- and PSD95-positive synaptic puncta in human neurons chronically treated with various Aβ peptides (**A**) representative images of human neurons immunolabeled for the dendritic marker MAP2 (blue), the presynaptic marker synapsin-1 (red), and the postsynaptic marker PSD95 (green), and additionally stained with the nuclear marker DAPI (cyan); for each image, a zoomed-in dendritic segment marked by a box is shown on the right; (**B**) summary graphs of the synapse density measured as puncta that are positive for both synapsin and PSD95; (**C** and **D**) summary graphs of the sizes of synapsin- (**C**) and PSD95-positive (**D**) puncta. (**E** and **F**) Density of synapsin- and PSD95-positive synaptic puncta in neurons treated with control Aβ40 and Aβ42 peptides composed of scrambled or reverse sequences. (**G**–**N**)STED super-resolution microscopy analysis of the effect of different concentrations of synthetic Aβ42 peptide on the presynaptic vesicle cluster visualized by staining for synapsin and PSD95. (**G**) Representative images of synapses stained for synapsin-1 and PSD95. (**H** and **J**) Summary graphs of the size of synapsin-positive (**H**) and PSD95-positive puncta (**J**). (**I** and **K**) Staining intensity of the synapsin-positive (**I**) or PSD95-positive puncta (**K**). (**L**) Representative images of synapses stained for synaptophysin and PSD95. (**M** and **N**) Summary graphs of size (**M**) and staining intensity (**N**) or synaptophysin-positive puncta. All neurons were analyzed at DIV45; all numerical data are mean ± SEM; numbers of experiments are reported in the bars (*n* = 3–4). Statistical significance was assessed by 1-way ANOVA with post hoc corrections and comparing the mean of each group with control (Ctrl). *P < 0.05, **P < 0.01, ***P < 0.001, and ****P < 0.0001. Nonsignificant comparisons are not indicated. Scale bars: 10 μm (**A** and **E**); 0.5 μm (**G** and **L**).More representative images and traces as well as analyses of the same data of pseudo-replicates instead of true replicates are shown in Supplemental Figure 4.

The Aβ peptide toxicity observed here and elsewhere (11–16) appears to contradict our own previous studies, which suggested that, at physiological levels in human neurons, Aβ peptides are synaptogenic instead of toxic (18). To address this apparent contradiction, we measured the density of synapses in human neurons under exactly the same conditions of chronic Aβ peptide treatments as used for the toxicity assays (Figure 1 and Supplemental Figure 1). For this purpose, we stained the treated neurons for the presynaptic marker Synapsin-1 and the postsynaptic marker PSD95 and measured the density and size of synaptic puncta that are positive for both markers, thereby enabling a rigorous assessment of synapse numbers and synapse dimensions (Figure 4, A–D, and Supplemental Figure 4, A–D).

Strikingly, Aβ40 significantly increased the density of synapses at all concentrations except for the lowest concentration, with a 2-fold maximum elevation of synapse numbers (Figure 3, A and B, and Supplemental Figure 3, A and B). No major changes in the sizes of synapsin- or PSD95-positive puncta were detected (Figure 3, C and D, and Supplemental Figure 3, C and D). Treatments with Aβ42, conversely, produced a different concentration-dependent effect: at low Aβ42 concentrations, we observed a small but significant increase in synapse density (approximately 25%) similar to the effect of Aβ40, whereas at high Aβ42 concentrations, we detected a dramatic loss of synapses (approximately 35%) (Figure 4, A and B, and Supplemental Figure 4, A and B). Moreover, at low Aβ42 concentrations the sizes of synapsin- but not PSD95-positive puncta were increased (approximately 25%), whereas at higher Aβ42 concentrations the sizes of the synapsin-positive puncta, but again not of the PSD95-positive puncta, were decreased (greater than 50%) (Figure 4, C and D, and Supplemental Figure 4, C and D). Thus, the surviving synapses at higher Aβ42 concentrations appear to contain smaller synaptic vesicle clusters that are labeled by the synapsin antibodies, but not smaller synaptic junctions as monitored by PSD95 antibodies. The effects of Aβ42^arctic^ on synapse densities and synaptic puncta sizes were similar to those of Aβ42, except that lower concentrations of Aβ42^arctic^ did not increase synapse numbers and that the loss of synapses at higher concentrations (greater than 55%) was larger than that observed for Aβ42 (Figure 4, A–D, and Supplemental Figure 4, A–D).

To verify that the synaptic phenotypes depend on the native amyloid sequence, we repeated the full concentration series with scrambled and inverted-sequence versions of Aβ40 and Aβ42. Across every dose tested, these control peptides left synapse density and puncta size indistinguishable from vehicle, showing neither the synaptogenic rise at low concentrations nor the synaptotoxic decline at high concentrations (Figure 4, E and F, and Supplemental Figure 4, E and F). These negative controls demonstrate that the effects attributed to native Aβ40 and Aβ42 are sequence specific rather than a general consequence of peptide exposure.

### Aβ peptide–induced synapse changes.

The toxicity we observed with Aβ42 peptides is accompanied by a massive decrease in synapse numbers and a change in synapse organization even though dendrites and endosomes are not noticeably affected (Figure 3 and Supplemental Figure 3) and the amount of cell death is small (Figure 1E and Supplemental Figure 1, E–G). These findings are consistent with a gradual toxicity process in which synapses are more vulnerable to the toxic Aβ insult than other parts of the neurons (2, 22, 23). Note that the lack of a toxic effect by Aβ40 does not imply that Aβ40 is generally not neurotoxic, since it seems likely that it is the oligomeric species of all Aβ peptides that is toxic. Under our precisely controlled conditions, Aβ40 is probably not incubated for a sufficiently long time to form aggregates or the nucleating effect of Aβ42 is missing, thus rendering it nontoxic (Figure 2, A–D, and Supplemental Figure 2, A–F) (24).

The decreases in the sizes of synapsin-positive but not of PSD95-positive synaptic puncta as a function of Aβ42 treatments is surprising because it suggests that Aβ42 toxicity does not just eliminate entire synapses but first impairs the presynaptic vesicle cluster. To confirm this hypothesis, we analyzed synapses by STED super-resolution microscopy (Figure 4, G and L, and Supplemental Figure 4, G and L). STED imaging confirmed the increase of the synapsin-positive puncta size at low Aβ42 concentrations (approximately 30%) and the large decrease in the size of synapsin-positive puncta at higher Aβ42 concentrations (approximately 70%) (Figure 4, G and H, and Supplemental Figure 4, G and H). Moreover, quantifications of the synapsin-staining intensity of the synapsin-positive puncta uncovered a large increase in synapsin-staining intensity (approximately 35%), mirroring the decrease in the sizes of the synapsin-positive puncta at high Aβ42 concentrations (Figure 4I and Supplemental Figure 4I). Together, these results suggest that the synaptic vesicles stained by the synapsin antibodies in the synapses that remain during the toxic assault by Aβ42 are contracting into tighter clusters. To strengthen these conclusions, we extended the analysis to an additional vesicle marker, Synaptophysin (25), and to the postsynaptic protein PSD95 using the same STED approach. Synaptophysin-positive boutons behaved exactly like the synapsin puncta, enlarging at low Aβ42 and contracting at synaptotoxic concentrations, whereas the area of the PSD95 specializations remained essentially constant across all conditions (Figure 4, J–N, and Supplemental Figure 4, J–N). The concordance of 2 independent presynaptic markers together with the stability of the postsynaptic compartment indicates that Aβ42 aggregates cause a selective collapse of the vesicle cloud without immediately altering the synaptic junction. Overall, our findings are consistent with the notion that low Aβ42 concentrations promote synapse formation whereas higher concentrations produce synapse toxicity associated with a reorganization of the presynaptic terminals. Since the changes in synapses observed are present in all neurons (Figure 4), whereas toxicity appears to affect some neurons more than others (Figure 1), it is likely that synaptotoxicity is more general after chronic Aβ aggregate exposure than overall neurotoxicity.

### Aβ control of neural network activity.

**Figure 5:**
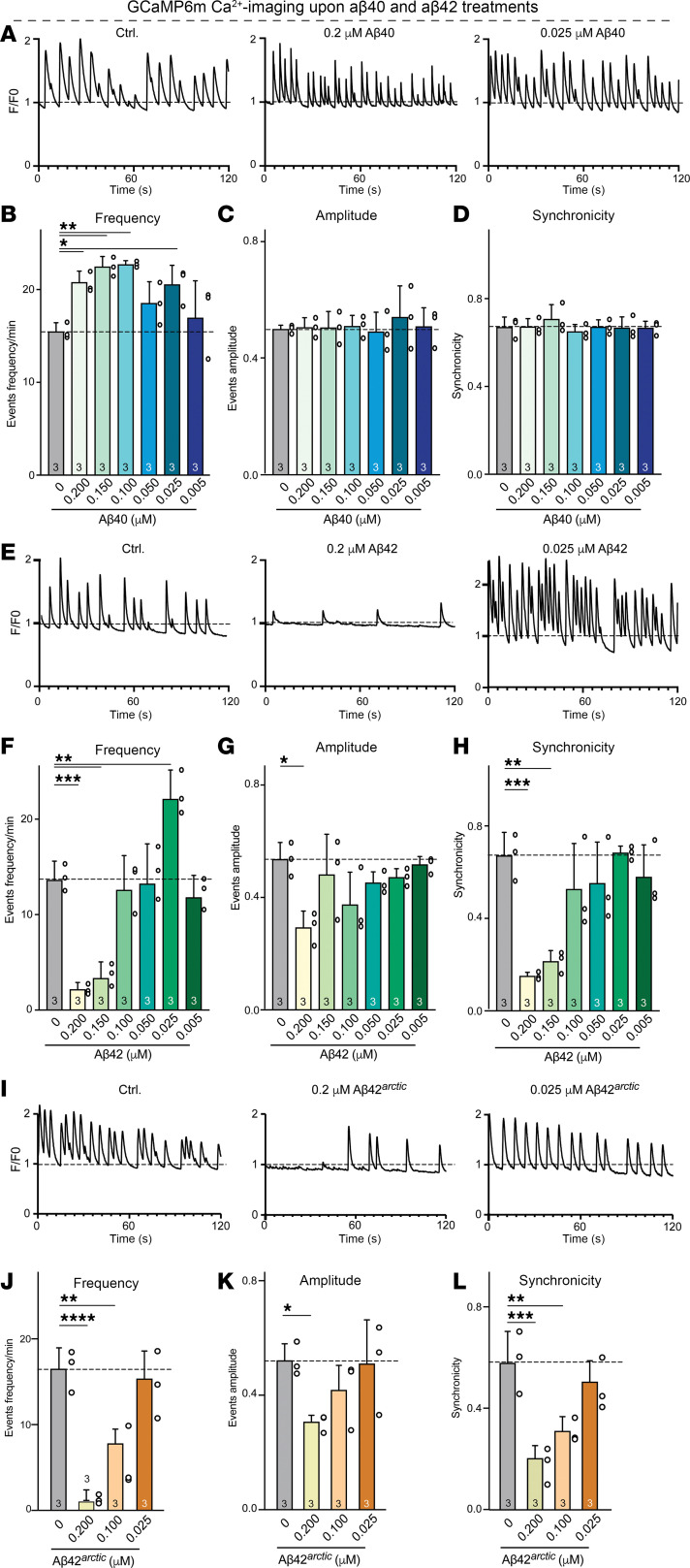
High concentrations of Aβ40 peptides enhance the network activity of human neurons as monitored by Ca^2+^-imaging, whereas high concentrations of Aβ42 and Aβ42^arctic^ peptides impair network activity. (**A–L**) Ca^2+^-imaging of human neurons chronically treated with synthetic Aβ40, Aβ42 or Aβ42^arctic^ peptides reveals increased network activity observed at all concentrations of Aβ40 and at low concentrations of Aβ42, but a deterioration of network activity induced by high concentrations of Aβ42 and Aβ42^arctic^. Human neurons expressing GcaMP6m under the control of the synapsin-1 promoter were treated as described in Figure 1A and imaged at DIV45 (**A**, **E**, and **I**, representative traces; **B**–**D**, **F**–**H** and **J**–**L**) summary graphs of the indicated parameters of network activity. All neurons were analyzed at DIV45; all numerical data are mean ± SEM; numbers of experiments are reported in the bars (*n* = 3). Statistical significance was assessed by 1-way ANOVA with post hoc corrections and comparing the mean of each group with control (Ctrl), with *P < 0.05, **P < 0.01, ***P < 0.001, and ****P < 0.0001. Nonsignificant comparisons are not indicated. More representative images and traces as well as analyses of the same data as pseudo-replicates instead of true replicates are shown in Supplemental Figure 5.

Are the structural changes we observed in synapses as a function of Aβ peptide treatments functionally relevant? To address this question, we performed Ca^2+^ imaging that enables monitoring network activity in cultured human neurons, which in turn depends on synaptic transmission. We infected human neurons with a lentiviral vector expressing gCaMP6m under the control of the human synapsin-1 promoter and recorded spontaneous Ca^2+^ transients as a measure of neuronal network activity at DIV45 (Figure 5, A, E, and I, and Supplemental Figure 5, A, E, and I) (26).

Neurons chronically treated with Aβ40 across all, except for the lowest, Aβ40 concentrations exhibited a greatly increased frequency of Ca^2+^ spikes compared with controls, without changes in Ca^2+^ spike amplitudes or synchronicity (Figure 5, A–D, and Supplemental Figure 5, A–D). This effect was more pronounced at higher concentrations of Aβ40, corresponding to the observed increase in synaptic puncta density and presynaptic size (Figure 4, A–D, and Supplemental Figure 4, A–D). Similarly, neurons treated with a synaptogenic, low concentration of Aβ42 (0.025 μM) also showed a significant elevation in Ca^2+^ transient frequency without changes in event amplitude or synchronicity (Figure 5, E–H, and Supplemental Figure 5, E–H). Neurons exposed to higher concentrations of Aβ42 (greater than or equal to 0.1 μM), however, displayed a significant decrease in Ca^2+^ transient frequency, amplitude, and synchronicity (Figure 5, F–H, and Supplemental Figure 5, F–H). The effect of higher Aβ42 concentrations was most pronounced for the network synchronicity in that Aβ42 seemed to almost completely abolish synchronous firing of the neurons, consistent with toxicity (Figure 5H and Supplemental Figure 5H. In agreement with its failure to promote synapse formation, Aβ42^arctic^ peptide did not enhance neuronal network activity at any concentration (Figure 5I and Supplemental Figure 5I). At the lowest doses, responses to the Aβ42^arctic^ peptide were indistinguishable from vehicle, while at concentrations that proved synaptotoxic, the cultures showed a pronounced fall in spike frequency and amplitude together with a near-complete breakdown of synchrony (Figure 5, J–L, and Supplemental Figure 5, J–L). These functional data mirror the structural findings on synapses (Figure 4) and reinforce the conclusion that aggregated Aβ42 species, including the Aβ42^arctic^ variant, compromise both synapse integrity and network function.

## Discussion

Our results reconcile 2 seemingly contradictory observations in the literature. On the one hand, numerous studies demonstrate that Aβ peptides elicit potent synapto- and neurotoxic effects (27–32). On the other hand, Aβ peptides also seem to enhance synapse formation (18). By using chemically defined synthetic Aβ peptides and by meticulously controlling the aggregation state and concentrations of these Aβ peptides, we have resolved this apparent contradiction. Specifically, we confirm earlier conclusions that higher concentrations of Aβ peptides are deleterious to neurons and their synapses (Figures 1, 4, and 5) (27–32), but also show that at low levels Aβ peptides present in a non-aggregated state are synaptogenic (Figures 4 and 5). Our data, therefore, suggest a simple yet compelling hypothesis: Free Aβ peptides serve a beneficial role at synapses whereas aggregation renders Aβ peptides pathogenic. High-resolution STED imaging provided a mechanistic foothold on what happens when Aβ peptides become synaptotoxic, revealing that synaptotoxic concentrations of Aβ42 compress the vesicle cloud into a tight disc at the active zone while leaving the PSD-95–defined postsynaptic density unchanged. Imaging for Synaptophysin mirrored the Synapsin pattern, pointing to a selective contraction of presynaptic vesicle clouds. This architectural shift was accompanied by functional failure. Together, these findings support the notion that soluble Aβ peptides act at physiological concentrations as neuronal modulators that enhance synaptic connections and accelerate network throughput. Once aggregation occurs, the same Aβ peptides become synaptotoxic agents that induce tighter clustering of synaptic vesicles at the presynaptic active zone, lower the presynaptic release probability, desynchronize firing, and subsequently breach the membrane to trigger synapse loss followed by neuronal cell death.

Note that our observations do not imply that Aβ40 is intrinsically benign. Instead, we deliberately designed our experiments to ensure that Aβ40 would not significantly aggregate under our conditions, which minimized its potential toxicity. Since Aβ40 is well known to aggregate more slowly than Aβ42 (Figure 2, A–D, and Supplemental Figure 2, A–F) (25), it remains entirely plausible that, given adequate time and conditions, Aβ40 aggregates could be just as synaptotoxic and neurotoxic as their Aβ42 counterparts. The critical transition between the synaptogenic and synaptotoxic actions of Aβ peptides thus is not between Aβ40 and Aβ42, but between free and aggregated forms of Aβ peptides. Indeed, the notion that all types of Aβ peptides carry the capacity for harm once aggregation initiates is consistent with a broad body of earlier work (27–32). Our fractionation experiments strengthened this conclusion: the pellet containing only large aggregates and the supernatant enriched in free Aβ peptides and smaller oligomers produced indistinguishable toxicity, suggesting that this toxicity is not caused by the free Aβ peptides or by large Aβ aggregates but by species in an intermediate state of aggregation (Supplemental Figure 2, G–I).

Our data establish that the native amino acid sequence of Aβ peptides is indispensable for both their synaptogenic and their synaptotoxic effect. Scrambled or inverted sequence peptides were inert in toxicity assays and left the synapse density unchanged at every dose, indicating that neither the synaptogenic nor the synaptotoxic action of Aβ peptides can be ascribed to their generic physicochemical properties. This observation highlights a previously underappreciated facet of Aβ peptide biology, namely that Aβ peptides may actually assume an essential physiological function when maintained in a free form.

Therapeutically, our results bolster current strategies aimed at neutralizing aggregated Aβ, as pursued by many ongoing clinical trials (4–6). Yet, our results also suggest that simply clearing all Aβ from the brain may be insufficient as an AD therapy and could even be counterproductive. In clinical trials where the elimination of aggregated plaques was significant, cognitive decline persistently continued (4–6). At the same time, in these trials, increased free Aβ42 levels in the CSF correlated with better therapeutic outcomes (17). Such findings echo our observations by underscoring a potential protective or supportive role for free Aβ. We thus hypothesize that Alzheimer’s disease pathology is propelled not merely by the presence of aggregates but also by a depletion of free Aβ that occurs when Aβ peptides become sequestered into increasingly larger aggregates (33). Accordingly, AD treatments may need to preserve the levels of free Aβ in patients to sustain the synaptogenic capacity of Aβ in addition to combating Aβ aggregates to limit the synaptotoxicity of Aβ. Current clinical findings lend support to this view. The approved Aβ immunotherapies (aducanumab, lecanemab, donanemab) (6, 34, 35) were designed to recognize aggregated forms and spare monomeric Aβ, consistent with our conclusion that neutralizing aggregates is desirable. Conversely, solanezumab, an antibody that preferentially bound monomeric Aβ, failed to yield cognitive benefit, a result that may reflect the unwanted removal of the very species needed for synaptic support (36).

A similarly dual function pattern is emerging with secretase modulation. Potent BACE1 inhibitors that drastically lowered total Aβ worsened cognition in late-stage trials, again consistent with the hazards of removing too much soluble peptide, although other explanations are also plausible (37). By contrast, modest BACE1 suppression appears compatible with normal cognitive performance, presumably because enough Aβ remains to sustain synaptic activity while aggregate formation is slowed (38). Taken together, the clinical and experimental evidence point toward a therapeutic sweet spot: aggregated Aβ must be neutralized or cleared, yet a physiological supply of soluble peptide should be preserved. Our data provide a mechanistic rationale for this balanced approach and a cellular benchmark for judging its success.

Finally, we would like to explicitly discuss some limitations of our study and the new questions it raises. All experiments were performed with human neurons cocultured on mouse glia; the synaptogenic actions of Aβ peptides cannot easily be probed in mouse cultures because they do not survive long enough and are challenging to investigate in a mouse brain in vivo because homeostatic mechanisms likely kick in rapidly. Moreover, we do not know the mechanisms mediating either the synaptogenic or synaptotoxic effects of Aβ peptides. The fact that inverted-sequence Aβ peptides are inactive suggests that they act with specificity and not, for example, via their detergent-like properties. A receptor seems likely to be involved, but which receptor might be responsible is unknown. Indeed, as of 2025 more than 6,000 papers refer to Aβ receptors and more than 10 receptors have been described, some of which are reviewed by Smith and Strittmatter (39) and Ng (40). Furthermore, although our findings document a robust toxic effect of Aβ peptide aggregates on synapses, our results do not reveal whether the Aβ aggregates may also be toxic for other components of our neuron-glia cultures. Future experiments will have to address these tantalizing questions.

## Methods

### Sex as a biological variable.

Sex was not evaluated as a biological variable. Both male and female CD1 mice aged postnatal days 0 to 2 (P0–2) were used in this study.

### Study design.

The current study individually examines the 2 most abundant Aβ peptides (Aβ40 and Aβ42), one super-aggregating mutant Aβ peptide that is linked to familial AD (Aβ 42^arctic^) and other peptides containing scrambled or inverted sequences of Aβ42 and Aβ40 as chemically defined synthetic peptides to assess their effects on neurons. The study was designed to take into account the differential propensity for aggregation of Aβ peptides that occurs in aqueous solutions in a temperature- and time-dependent manner. For this purpose, the formulation and timing of Aβ administrations were precisely scripted. For all experiments, at least 3 biological replicates (independent experiments) were performed. Our data are represented both as true replicates using the number of experiments as the ‘n’ (shown in the main figures) and as pseudoreplicates using the number of neurons or ROIs as the ‘n’ for statistical analysis (Supplemental figures). All analyses were performed out in a ‘blinded’ fashion whereby the experimenter was unaware of the sample identity.

### Mouse lines.

WT female and male CD1 mice aged postnatal days 0 to 2 (P0–2) were used for primary glia cultures, with cells pooled from the 2 sexes. The mice were housed in the Stanford SIM1 animal facility under the supervision of the Stanford animal care unit; all animals were healthy and had not participated in previous experiments. All animal experiments were reviewed and approved by the Stanford IACUC.

### HEK293T cell lines.

HEK293T cells were purchased from ATCC and low-passage cells were expanded and stored. HEK293T cells below passage 20 were cultured in DMEM (Gibco) + 10% fetal bovine serum (FBS) (Sigma-Aldrich) and reached confluency every 3 days, at which point they were passaged (1:20 split ratio).

### ES cell cultures.

Male human embryonic stem cells (ESC), line H1, were obtained from WiCell (line WA01). The stem cells were maintained feeder-free in mTeSR1 medium (Stem Cell Technologies), from frozen cell stocks of passage 50. With each passage, cells were detached with Accutase at 37°C, centrifuged and resuspended in mTeSR1 with 2 μM thiazovivin (BioVision), and then replated on Matrigel-coated 6-well plates. The Stem Cell Research Oversight (SCRO) at Stanford University approved the protocols used in this work (SCRO 518).

### Glial cell culture.

Mouse glial cells were prepared from the cortices of newborn CD1 pups, which were dissected in Hanks′ Balanced Salts (HBSS) at postnatal days 0 to 2 (P0–2). The cortex was digested with 80 μL of papain in 5 mL of HBSS for 20 minutes at 37°C. After digestion, the tissues were washed 3 times with DMEM (Gibco) supplemented with 10% FBS (Sigma-Aldrich). The cells were then vigorously triturated and plated into T75 cell culture flasks containing 12 mL of DMEM with 10% FBS. Once the glial cultures reached approximately 90% confluence — about seven days after dissection — they were detached using 0.05% trypsin and replated into three new T75 flasks at a 1:3 split ratio in DMEM with 10% FBS. This replating step and the specific media used prevent the survival of mouse neurons, resulting in mixed glia cultures. No antibiotics were added to the cultures (41).

### Virus generation.

All lentiviruses used in this study were produced as previously described (41). Generally, lentiviral vectors were cotransfected into HEK293T cells (ATCC) using calcium phosphate and HBS (Takara), with 3 helper plasmids (pRSV-REV, pMDLg/pRRE, and vesicular stomatitis virus G protein expression vector (VSV)). In total, 12 μg of lentiviral packaging DNA were transfected per T75 flask of HEK293T cells: Rev (4 μg), RRE (8 μg), and VSV (6 μg). Forty-eight hours after transfection, cell media was harvested and centrifuged at 19,000*g* for 2 hours at 4°C. Pellets were resuspended overnight at 4°C in 100 μl of DMEM (Gibco), aliquoted, and frozen at –80°C.

### Generation of human neurons.

Ngn2-iN cells (human neurons) were generated as previously described (42). hES cells were treated with Accutase, plated as dissociated single cells on Matrigel-coated plates, and infected with Ngn2 and rtTA lentiviruses in mTeSR with thiazovivin. The next day (DIV1), the culture medium was replaced with DMEM/F12 (Gibco) with addition of N2 (STEMCELL Technologies), NEAA, BDNF (10 ng/ml, PeproTech), human NT3 (10 ng/ml, PeproTech), mouse Laminin-1 (0.2 μg/ml, PeproTech) and 2 μg/ml doxycycline to induce Ngn2 expression. On DIV 2 and DIV 3, puromycin (1 μg/ml) was used to select infected cells. On DIV 5, iN cells were dissociated using Accutase and plated on mouse glial cells growing on coverslips at 100,000–150,000 cells/well in 24-well plate in the Neurobasal A medium (Gibco) with B27 (Gemini21) (GeminiBio), doxycycline, BDNF (PeproTech), NT3 (PeproTech), Laminin-1 (PeproTech), 5% FBS (Sigma-Aldrich), and GlutaMAX (Thermo-Fisher). Cell medium was half changed every other day until DIV 10, then once a week until DIV 45. At this time point, cells were fixed for immunocytochemistry or used for live imaging analyses.

### Primary neuron culture.

At DIV0, Hippocampi were dissected from P0 mice, digested by papain (Worthington) for 20 minutes at 37°C, filtered through a 70 μm cell strainer (Falcon), and plated on 0.1 mg/mL poly-D-lysine (Gibco) coated coverslips in 24-well plates. Plating media contained 5% FBS (Sigma-Aldrich), B27 (Invitrogen), 0.4% glucose (Millipore-Sigma), and 2 mM glutamine (Gibco) in MEM (Gibco). After 1 hour, culture medium was changed to growth medium containing 5% FBS, B27 (Invitrogen), and GlutaMAX (Thermo-Fisher) in Neurobasal A (Gibco). On DIV4 and DIV8, half of the medium was changed by growth medium containing 4 μM Ara-C (Santa Cruz Biotechnology), and neurons were analyzed at DIV16 (26).

### Preparation of synthetic Aβ peptides.

Synthetic Aβ peptides (Aβ40, Aβ42, their scrambled and inverted sequences versions, and Aβ42^arctic^) were purchased in lyophilized form from Abcam Limited (Aβ40, Aβ42, and their inverted sequences versions) and Anaspec (Scrambled versions of Aβ40 and Aβ42, as well as Aβ42^arctic^). Peptides were initially dissolved in sterile-filtered dimethyl sulfoxide (DMSO) (Sigma-Aldrich) to create stock solutions at a concentration of 1 mg/mL. These stock solutions were then aliquoted and serially diluted with DMSO to achieve final concentrations of 0.2 μM, 0.15 μM, 0.1 μM, 0.05 μM, 0.025 μM, and 0.005 μM when added to the cell culture media. For scrambled and inverted sequences versions of Aβ40 and Aβ42, only 0.2 μM and 0.025 μM concentrations were prepared. To preserve the oligomerization state of the peptides, the diluted aliquots were snap frozen immediately after preparation by immersion in liquid nitrogen. For experimental blinding, an independent operator coded the aliquots so that the experimental operator was unaware of the peptide identity and concentration during assays. The aliquots were stored at –80 °C until use. Each aliquot was used only once, and 1 vial of peptide obtained from Abcam or Anaspec was used per experiment.

### Administration of Aβ Peptides to human neurons.

The prepared aliquots of Aβ peptides were added directly to the culture media during each routine half-media change. To support optimal neuronal development, maturation, and function, while preserving essential factors released by neurons and glia, fresh media supplemented with Aβ was provided every other day until DIV10, and then once weekly until DIV45. This half-media change protocol ensured the retention of endogenous neurotrophic factors important for neuronal health (42). During each media change, the Aβ peptides were introduced into the cultures to achieve final concentrations of 0.2 μM, 0.15 μM, 0.1 μM, 0.05 μM, 0.025 μM, and 0.005 μM. For scrambled and inverted sequence peptides, only the 0.2 μM and 0.025 μM concentrations were tested. At DIV6, we initiated a half-media replacement protocol in which 50% of the old culture medium was carefully removed and replaced with fresh medium containing Aβ peptides at twice the intended final concentration. Because the remaining 50% of the medium at DIV6 contained no peptide, this 1:1 mixing resulted in the target final concentration across the entire well. For example, to achieve a final concentration of 0.2 μM, the fresh medium added at DIV6 was prepared at 0.4 μM, yielding a uniform 0.2 μM concentration after mixing with the peptide-free half. Starting from DIV8 and onward, subsequent half-media changes replaced half of the existing medium with fresh medium containing Aβ at the same target concentration (e.g., 0.2 μM), since the remaining half already contained approximately that amount. This approach was designed to mimic a chronic treatment regimen, allowing us to investigate the long-term effects of the peptides on synapse formation and neuronal activity under sustained exposure conditions.

### Administration of Aβ Peptides to primary neurons.

Aβ peptides were added to the cell culture media during routine half-media changes as explained for human neurons. Specifically, at DIV2, DIV4, DIV8, and DIV14, 50% of the existing culture medium was carefully removed from each well. The freshly prepared medium containing Aβ40, or Aβ42, at final concentrations of 0.2 μM, 0.1 μM, or 0.025 μM was then added in an equal volume, ensuring consistent exposure to the peptides throughout the experimental period. Cells were maintained under these chronic treatment conditions until DIV16, at which point they were subjected to subsequent analyses.

### TdTomato cytotoxicity assay.

To assess neuronal cytotoxicity induced by Aβ peptides, we employed a novel Td-Tomato cytotoxicity assay that we developed. Human neurons were infected at DIV 6 with a lentiviral vector carrying a plasmid encoding nuclear localization signal (NLS)-TdTomato under the control of the human Synapsin-1 promoter. The use of the Synapsin promoter ensured neuron-specific expression of Td-Tomato. In our human neurons, Synapsin expression becomes significant after 18–20 days in culture; therefore, Td-Tomato labeling in neuronal nuclei was weak before this time point. When the neurons had reached full maturation, cells were examined for Td-Tomato localization. Neurons displaying cytoplasmic Td-Tomato were counted as indicators of cytotoxicity. The assay was benchmarked at DIV30 with 2 reference insults, 5% DMSO and 200 μM H_2_O_2_, each applied for 4 hours; both triggered a robust nuclear-to-cytoplasmic shift, confirming the reporter’s sensitivity. For Aβ experiments, imaging and scoring were performed at DIV45, immediately after the final peptide exposure. Data were quantified by calculating the density of neurons with cytoplasmic Td-Tomato, normalized to control cultures, and presented graphically. To assess cell viability, the number of Td-Tomato–positive nuclei, which represent surviving neurons, was counted, normalized to control values, and reported in graphs.

### MTT cytotoxicity assay.

The MTT assay is a colorimetric method that evaluates cell viability, proliferation, and cytotoxicity by utilizing the yellow tetrazolium dye 3-(4,5-dimethylthiazol-2-yl)-2,5-diphenyltetrazolium bromide (MTT) (43–47). Metabolically active cells reduce MTT to purple formazan crystals through mitochondrial dehydrogenase activity, with the amount of formazan produced being proportional to the number of viable cells. We conducted the MTT assay on both treated and control human neurons at DIV45. MTT powder (Sigma-Aldrich) was reconstituted in 3 mL of balanced salt solution and stored at 4°C until use. As a positive control for cytotoxicity, cells were incubated with 10% DMSO for 24 hours prior to the assay. MTT solution was added to the culture media at an amount equal to 10% of the culture medium volume. The cultures were then returned to the incubator and maintained at 37°C for 4 hours to allow for formazan crystal formation. Following incubation, the culture media containing MTT was carefully removed, and the resulting formazan crystals were dissolved by adding an amount of MTT solubilization solution (Sigma-Aldrich) equal to the original culture medium volume. The absorbance of the solubilized formazan was measured at 570 nm using a spectrophotometer, with background absorbance at 690 nm subtracted from each reading to correct for nonspecific signals. Data were normalized to control values and presented graphically to illustrate the effects of Aβ peptide treatments on cell viability.

### Cell death assay.

To obtain a live cell read out of neuronal death, we monitored loss of plasma-membrane integrity with Incucyte Cytotox Green reagent (Sartorius), a cell-impermeant DNA dye that fluoresces upon binding to nuclear DNA once the membrane is breached (48). A 250× stock supplied by the manufacturer was thawed on ice and diluted into prewarmed culture medium to a final working concentration of 250 nM immediately before use. For each assay plate, half the medium was removed and replaced with dye-containing medium so that the final dye concentration and vehicle composition were identical across all conditions. For validation, DIV30 cocultures were challenged for 4 hours with 200 μM H_2_O_2_; fluorescence images were acquired immediately thereafter. For Aβ experiments, cultures had received chronic peptide exposure from DIV6 onward and were analyzed at DIV45. Dye was present for 2 hours before imaging to allow equilibrium without inducing additional stress. Ten nonoverlapping fields per well were captured with the 20× objective using green fluorescence channels. Exposure settings were kept constant within an experiment. Images were processed with ImageJ. A top-hat background subtraction was applied to the green channel, and a fluorescence threshold was kept constant across batches. The number of green-positive nuclei was expressed either as absolute counts or normalized per control.

### Immunofluorescence analysis.

Mature human iN cells were washed 3 times with PBS (Gibco) and fixed with 4% PFA (Electron Microscopy Science) and 4% sucrose (Sigma-Aldrich) in PBS for 10 minutes at room temperature. Cells were then washed three times with PBS, followed by permeabilization and blocking with 0.25% Triton-X-100 (Sigma-Aldrich), 2.5% bovine serum albumin (BSA) (GeminiBio) and 2,5% NGS (Sigma-Aldrich) for one hour at room temperature. Incubation with primary antibodies was performed overnight at 4°C in the corresponding blocking buffer (41). For endosome analyses, fixing solution contained only 4% PFA and the permeabilization was performed separately before the blocking step, using 0.1% Triton-X-100. Primary Antibodies used include rabbit monoclonal anti-Synapsin-1 (Yenzym antibodies, clone YZ6078, 1:1000 dilution), mouse monoclonal anti-PSD95 (Thermo Fisher Scientific, 7E3-1B8, 1:250 dilution), chicken polyclonal anti-MAP2 (EnCor, CPCA-MAP2, 1:1000 dilution), mouse monoclonal 6E10 (BioLegend, SIG-39320, 1:1000), rabbit monoclonal anti-Synaptophysin (homemade, P580 (26)), and rabbit monoclonal anti-EEA1(Cell Signaling, C45B10, 1:1000 dilution). Cells were washed 3 times with PBS and incubated for 1 hour at room temperature with Alexa conjugated secondary antibodies: goat anti-chicken Alexa 647, goat anti-rabbit Alexa 546, goat anti-rabbit Alexa 488, and goat anti-mouse Alexa 488, all 1:500. Coverslips were washed once with PBS and once with water to remove salts before mounting, then were mounted using Fluoromount (with DAPI) (Thermo Fisher Scientific, 00-4959-52) on glass slides. All images were taken with the Nikon A1RSi confocal microscope (Nikon Instruments Inc.) for analysis of synaptic puncta using Nikon Analysis Software.

### Quantification of 6E10-positive aggregates.

To quantify the density and size of 6E10-immunopositive aggregates, we acquired high-resolution confocal images at 60x magnification using a Nikon Eclipse Ti microscope (Nikon, Japan). All fluorescence parameters, including laser power and detector settings, were held constant throughout image acquisition to ensure comparability across samples. We then processed and analyzed the resulting images with Nikon Analysis Software, employing standardized thresholds for object detection. For analyses conducted in human neurons, the density of 6E10-positive aggregates was determined by calculating the number of discrete objects per unit area of the imaged field. In contrast, for analyses in primary neurons, we recorded the total number of discrete objects within each field. In both cases, we measured particle size as the fraction of the total field occupied by 6E10-positive signals, using a binary region of interest encompassing the entire field of view. This approach allowed for consistent, unbiased quantification of both the distribution and relative size of immunofluorescent aggregates under different experimental conditions.

### Aβ42 fractionation experiment.

Aβ42 stock solutions were incubated at 37°C for 24 hours to generate a population of assemblies. Aggregation mixtures were centrifuged at 20,000*g* for 15 minutes at 4°C. The supernatant (enriched in monomer, small oligomers and residual larger species) was removed without disturbing the pellet. The pellet (large aggregates and high-molecular-weight oligomers) was resuspended in DMSO to the original volume. Supernatant and the resuspended pellet were administered to neurons, independently, at the concentration of 0.2 μM following the same protocol of administration used in this study (Figure 1A) and described above.

### Cell morphology.

Confocal imaging was conducted at 20× magnification. MAP2-positive dendritic arborization and soma size were quantified by semiautomated analysis with the SNT plugin from ImageJ (National Institutes of Health).

### Somatic endosomes.

All images were acquired at 60X magnification. Endosomes EEA-1–positive were analyzed by automatic particle counting from ImageJ (National Institutes of Health).

### Quantification of synaptic puncta.

The density, size, and staining intensity of synaptic puncta where pre- and postsynaptic marker signals (Synapsin-1 and PSD95) colocalized were quantified using Nikon Analysis Software. High-resolution fluorescence images at 60X were acquired using a Nikon (microscope model, e.g., Nikon Eclipse Ti). Consistent exposure settings were maintained across all samples to ensure comparability. For image analysis, regions of interest (ROIs) were selected within each image to encompass representative areas of the neuronal cultures, specifically focusing on secondary dendritic branches. Only colocalized puncta (where the signals from pre- and postsynaptic markers overlapped) were quantified. The close proximity of these markers was considered indicative of active synapses. The software’s automated detection tools were utilized to identify synaptic puncta based on fluorescence intensity thresholds. A uniform threshold level was applied to all images to distinguish specific staining from background noise effectively. Synaptic puncta density was calculated by counting the number of colocalized Synapsin-1 and PSD95-positive puncta within the ROI and normalizing it to the area of the ROI (puncta per μm²). The size of each synaptic punctum was determined by measuring its area (in μm²) as detected by the software. Intensity measurements were obtained by calculating the mean fluorescence intensity of the puncta, providing an indication of the expression levels of synaptic proteins (41). All image analyses were conducted in a blinded manner to eliminate observer bias. Data were collected from multiple independent experiments, and statistical analyses were performed to assess the effects of Aβ peptide treatments on synaptic parameters.

### STED super-resolution microscopy.

To investigate the alterations in presynaptic Synapsin-1 puncta size observed after Aβ42 treatment, we employed super-resolution microscopy using a Stimulated Emission Depletion (STED) microscope (Abberior Instruments GmbH). Human neuron cultures were immunostained for Synapsin-1, Synaptophysin, and PSD95, as previously described at *Immunofluorescence analyses*. Regions of interest (ROIs) specifically containing synapses were selected for imaging. High-resolution images were acquired at 100× magnification using an oil-immersion objective lens with a high numerical aperture, optimized for super-resolution imaging. Image acquisition was performed using the STED mode to achieve a resolution beyond the diffraction limit, allowing detailed visualization of synaptic structures. Following image capture, deconvolution was applied using Huygens Professional (Scientific Volume Imaging) to enhance image quality and resolution. The presynaptic Synapsin-1, Synaptophysin, and PSD95 signals were isolated by filtering out nonspecific staining and background noise. Quantitative analysis of Synapsin-1, PSD-95, and Synaptophysin puncta was conducted using ImageJ software (National Institutes of Health). The area occupied by Synapsin-1 staining within each punctum was measured by setting a consistent threshold to define puncta boundaries across all images. Measurements focused on puncta within the selected ROIs to ensure consistency. The size (area in μm²) of individual Synapsin-1, Synaptophysin, and PSD-95–positive puncta were calculated, and average puncta sizes were determined for each treatment group. Data were collected from multiple independent experiments to ensure statistical robustness. Statistical analyses were performed to assess the significance of changes in Synapsin-1, Synaptophysin, and PSD-95–positive puncta size due to Aβ42 treatment at various concentrations. All imaging and analyses were conducted in a blinded manner to eliminate observer bias.

### GCaMP6m live imaging recordings of Ca^2+^ signals.

To evaluate the effects of Aβ peptide treatments on neuronal network activity, live calcium imaging was performed using human neurons expressing the genetically encoded calcium indicator GCaMP. At DIV6, human neurons were infected with a lentiviral vector, prepared as described previously at *Virus generation*, carrying a plasmid encoding GCaMP6m under the control of the human Synapsin-1 promoter, ensuring neuron-specific expression. GCaMP6m fluorescence signal was first observed around DIV18–20, consistent with the activation of the Synapsin-1 promoter during neuronal maturation. At DIV45, when the neurons had reached full maturation, synaptic activity was recorded. For potentiating synaptic transmission of human neurons, the cells were imaged with ambient 4 mM CaCl_2_ and 8 mM KCl equilibrated in a HEPES-based buffer (140 mM NaCl, 10 mM HEPES, 20 mM glucose, 1 mM MgCl2). Live imaging was conducted using a Leica, model CTR6000, equipped with camera Ixon Ultra 897 and an incubation chamber to maintain optimal physiological conditions (37°C, 5% CO_2_) during recordings. Neuronal activity was captured in 2-minute videos at a frame rate of 10 frames/second, allowing for the detection of spontaneous calcium transients indicative of neuronal firing. Following acquisition, the videos were analyzed using MATLAB software (MathWorks). Regions of interest (ROIs) corresponding to 10–20 neurons were selected, and fluorescence intensity changes over time were extracted for each ROI. Analyses focused on quantifying the frequency, amplitude, and synchronicity of calcium signal peaks across the neuronal network. Signal peaks were identified using a peak detection algorithm, and synchronicity was assessed through cross-correlation analyses between neuronal pairs or by calculating network synchrony measures (26).

### Native SDS-PAGE.

To assess the aggregation state of Aβ peptides in stock solutions, we performed native polyacrylamide gel electrophoresis (PAGE) followed by immunoblotting. Stock solutions of Aβ peptides were incubated at 37°C for 1.5 and/or 1 hours to promote aggregate formation. After incubation, samples were diluted 1:2 with native PAGE sample buffer to maintain nondenaturing conditions. The samples were loaded onto 4%–15% gradient polyacrylamide Criterion gels (Bio-Rad), which allow for the separation of proteins across a wide range of molecular weights under native conditions. Electrophoresis was conducted using Tris-Glycine buffer at 4°C for 3 hours to preserve the native structure of the peptide aggregates during separation. A constant voltage appropriate for native PAGE was applied throughout the run (49). Following electrophoresis, proteins were transferred onto Nitrocellulose (0.45 μm) membranes using a semidry transfer apparatus optimized for proteins of mixed molecular weights. The transfer was performed according to the manufacturer’s instructions, ensuring efficient transfer of both low and high molecular weight species. Membranes were blocked with 5% BSA (GeminiBio) in Tris-buffered saline with 0.1% Tween-20 (TBST) at room temperature for 1 hour to prevent nonspecific antibody binding. After blocking, membranes were incubated overnight at 4°C with gentle agitation in primary antibody solution containing monoclonal mouse anti-β-Amyloid antibody (4G8 clone, BioLegend, anti-Aβ oligomers, ab126892, Abcam) diluted 1:1000 in TBST with 5% BSA. Following primary antibody incubation, membranes were washed 5 times for 5 minutes each with TBST to remove unbound antibody. They were then incubated with an appropriate secondary antibody conjugated to horseradish peroxidase (HRP), diluted in TBST, for 1 hour at room temperature with gentle agitation. After secondary antibody incubation, membranes were washed again as before. Blots were imaged using the Odyssey Infrared Imager CLX and analyzed using Image Study 5.2.5. (LI-COR Biosciences).

### Aβ42 fractionation validation.

Fractionation of Aβ42 was confirmed by blotting both fractions on a native PAGE gel, following the same protocol described above.

### Mass spectrometry.

To prepare samples for mass spectrometry analysis, Aβ peptides were dissolved in 0.1 M Triethylammonium bicarbonate (TEAB, Sigma) to reach the concentration of 1 mg/mL. MonoSpin C18 Solid-Phase Extraction (SPE) columns (GL Sciences) were equilibrated and washed 2 times with 200 μL of the equilibration solution containing 50% Acetonitrile (VWR) in water and wash solution containing 0.1% formic acid (Pierce) in water. 100 μg of the peptides were acidified by adding 50% formic acid to reach pH less than 4 and applied to the spin column. The samples were washed twice by adding 200 μL of the wash solution and then eluted by adding 200 μL of the elution solution containing 60% acetonitrile and 40% of 0.1% formic acid in water. Finally, the samples were dried via SpeedVac (ThermoFisher Scientific) and exchanged into LC-MS reconstitution buffer containing 2% acetonitrile with 0.1% formic acid in water for instrumental analysis. Aβ peptides were separated using an in-house pulled and packed reversed phase analytical column (approximately 25 cm in length, 100 microns of inner diameter), with Dr. Maisch 1.8 micron C18 beads as the stationary phase. Separation was performed with an 80-minute reverse-phase gradient (2%–45% B, followed by a high-B wash) on an Acquity M-Class UPLC system (Waters Corporation) at a flow rate of 300 nL/min. Mobile Phase A was 0.2% formic acid in water, while Mobile Phase B was 0.2% formic acid in acetonitrile. Ions were formed by electrospray ionization and analyzed either by an Orbitrap Exploris 480 mass spectrometer (Thermo Scientific), or an Orbitrap Eclipse Tribrid mass spectrometer (Thermo Scientific). The mass spectrometer was operated in a data-dependent mode using HCD (for Orbitrap Exploris 480) or CID (for Orbitrap Eclipse) fragmentation for MS/MS spectra generation. The raw data were analyzed using Byonic v5.1.1 (Protein Metrics) to identify peptides and infer proteins. A concatenated FASTA file containing Uniprot human proteins, bait sequences, and other likely contaminants and impurities was used to generate an in-silico peptide library. The precursor ion tolerance was set to 12 ppm. The fragment ion tolerance was set to 0.4 Da for data collected on Orbitrap Eclipse. Variable modifications included oxidation on methionine, histidine, and tryptophan, deamidation of glutamine and asparagine. Proteins were held to a false discovery rate of 1% using standard reverse-decoy technique (50).

### Statistics.

No statistical methods were used to predetermine sample size because effect sizes were unknown before experiments. Statistical significances for comparisons between treated groups and control were calculated using 1-way ANOVA and Dunnett’s post hoc in GraphPad Prism. All data in bar graphs and summary plots are shown as mean ± SEM. Numbers in bars represent the number of biological replicates or of pseudoreplicates, with statistical significance denoted by asterisks. *P* < 0.05 was considered statistically significant.

### Study approval.

All experimental procedures were approved by Stanford University’s Administrative Panel on Laboratory Animal Care (APLAC).

### Data availability.

All raw data, including images and recordings, are made publicly available at the Stanford Digital Repository without restrictions (https://doi.org/10.25740/hy621ks2628). Values for all data points in graphs are reported in the Supporting Data Values file.

## Author contributions

Conceptualization: AS, TCS. Methodology: AS, HS, TCS. Investigation: AS, SN (Data collection for the neuronal morphology and endosomes experiments), CHW (Data collection for the 6E10 analyses conducted on mouse primary neuronal cultures), HS (Mass spectrometry). Supervision: TCS. Writing – original draft: AS, TCS. Writing – review & editing: AS, TCS.

## Funding support

This work is the result of NIH funding, in whole or in part, and is subject to the NIH Public Access Policy. Through acceptance of this federal funding, the NIH has been given a right to make the work publicly available in PubMed Central.

- The NIH (AG070919 and AG048131 to TCS).
- The Greater Houston Community Foundation (to TCS).

## Supplementary Material

Supplemental data

Supplemental data set 1

Unedited blot and gel images

Supporting data values

## Version 1 09/30/2025

In-Press Preview

## Version 2 12/15/2025

Electronic publication
