# Supplementary material for: Aggregation shifts amyloid-β peptides from synaptogenic to synaptotoxic
Source: J Clin Invest. 2025 Sep 30;135(24):e193407. doi: 10.1172/JCI193407 (PMC12700558; doi:10.1172/JCI193407)
Supplement: Supplemental data [file jci-135-193407-s228.pdf]

Supplementary Figures and **Supplementary Figure Legends**

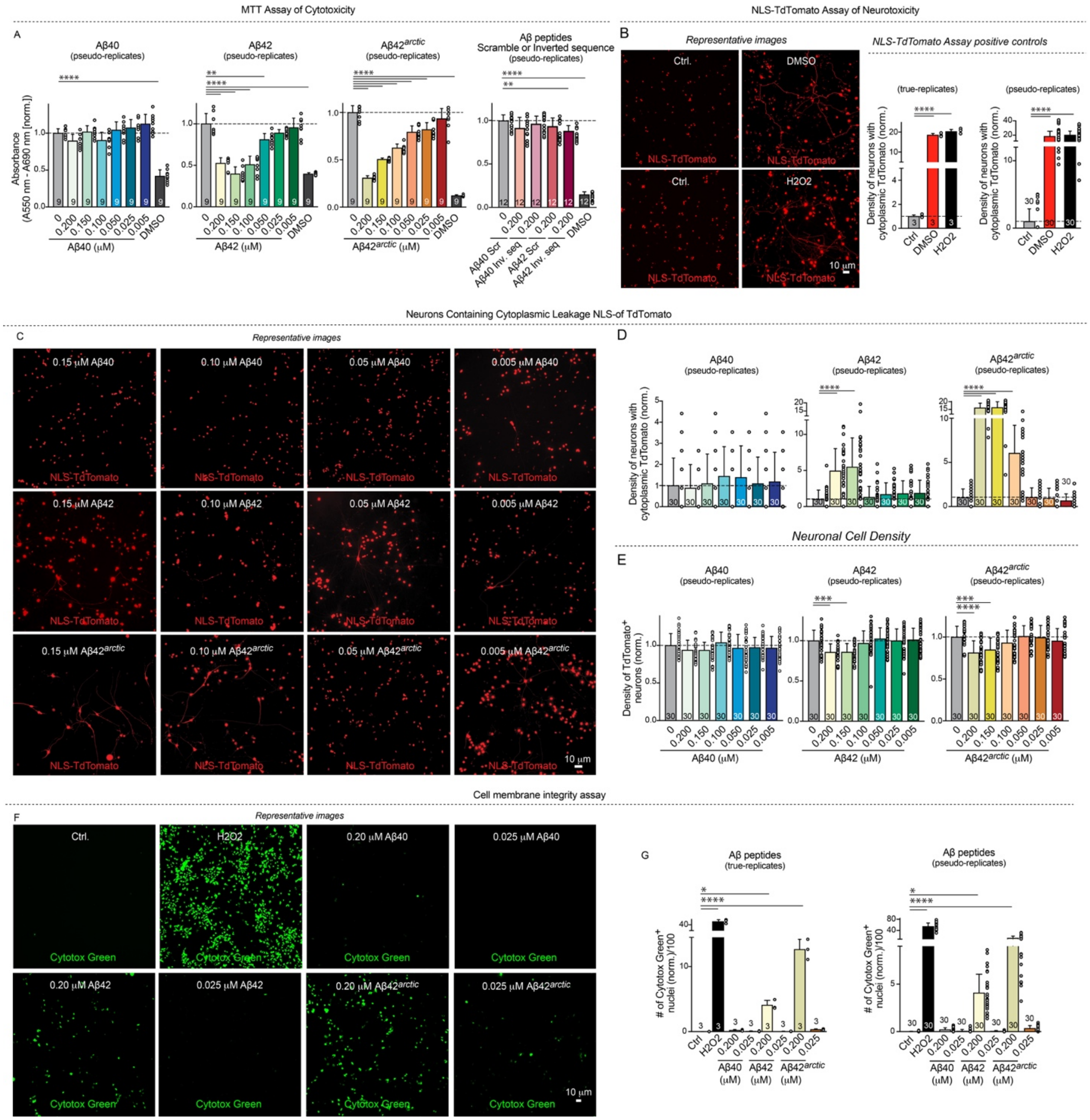

Supplementary Figure S1 | **MTT assays assessing the toxicity of Aβ40 and Aβ42, the scrambled and inverted-sequence versions of Aβ40 and Aβ42, and Aβ42^{arctic} peptides as in Figure 1 but using pseudo-replicates instead of true replicates (A), validation of the NLS-TdTomato nuclear localization assay illustrating neurotoxicity (B), additional images of the NLS-TdTomato nuclear localization assay (C), quantifications of Aβ-induced toxicity with the NLS-TdTomato assay using pseudo-replicate analyses (D & E), and cell death assayed using a membrane integrity assay (F & G)**

A. Quantification of the toxicity of Aβ40 and Aβ42, scrambled and inverted sequence versions of Aβ40 and Aβ42, and $Aβ42^{arctic}$ synthetic peptides using the MTT assay, but now analyzed as pseudo-replicates. Data are the same as in Figure 1B.

B. Validation of the NLS-TdTomato nuclear localization assay as a precise measure of neuronal toxicity using hydrogen peroxide as positive control. Data are presented both as true and as pseudo-replicates.

C. Additional representative images of human neurons expressing NLS-TdTomato showing conditions for which no images were included in Fig. 1C.

D. Quantification of the neurotoxicity of Aβ40, Aβ42, and $Aβ42^{arctic}$ synthetic peptides using the NLS-TdTomato nuclear localization assay, statistically analyzed as pseudo-replicates. Data are the same as those shown in Fig. 1D.

E. Quantification of the effect of Aβ40, Aβ42, and $Aβ42^{arctic}$ synthetic peptides on neuronal cell numbers, analyzed as pseudo-replicates. Data are the same as those shown in Fig. 1E.

F & G. Independent validation of the toxicity of Aβ40, Aβ42, and $Aβ42^{arctic}$ peptides using a third assay that measures the cell membrane integrity as an indication of cell death using a membrane-impermeable DNA dye (**F**, representative pictures displaying fluorescent nuclei; **G,** summary graphs displaying quantification of the labeled nuclei number). Data presented both as true and pseudo-replicates.

Human neurons used for the investigation were analyzed at DIV45; all numerical data are means ± SEM; numbers of experiments are reported in the bars (n=3); numbers of pseudo-replicates (n=9-30), intended as number of different non-overlapping field of view analyzed for each condition, are reported in the bars. Statistical significance for all the summary graphs in the figure was assessed by one-way ANOVA with post-hoc corrections and comparing the mean of each group with control (Ctrl), with *$p < 0.05$, **$p < 0.01$, ***$p < 0.001$, and ****$p < 0.0001$. Nonsignificant comparisons are not indicated.

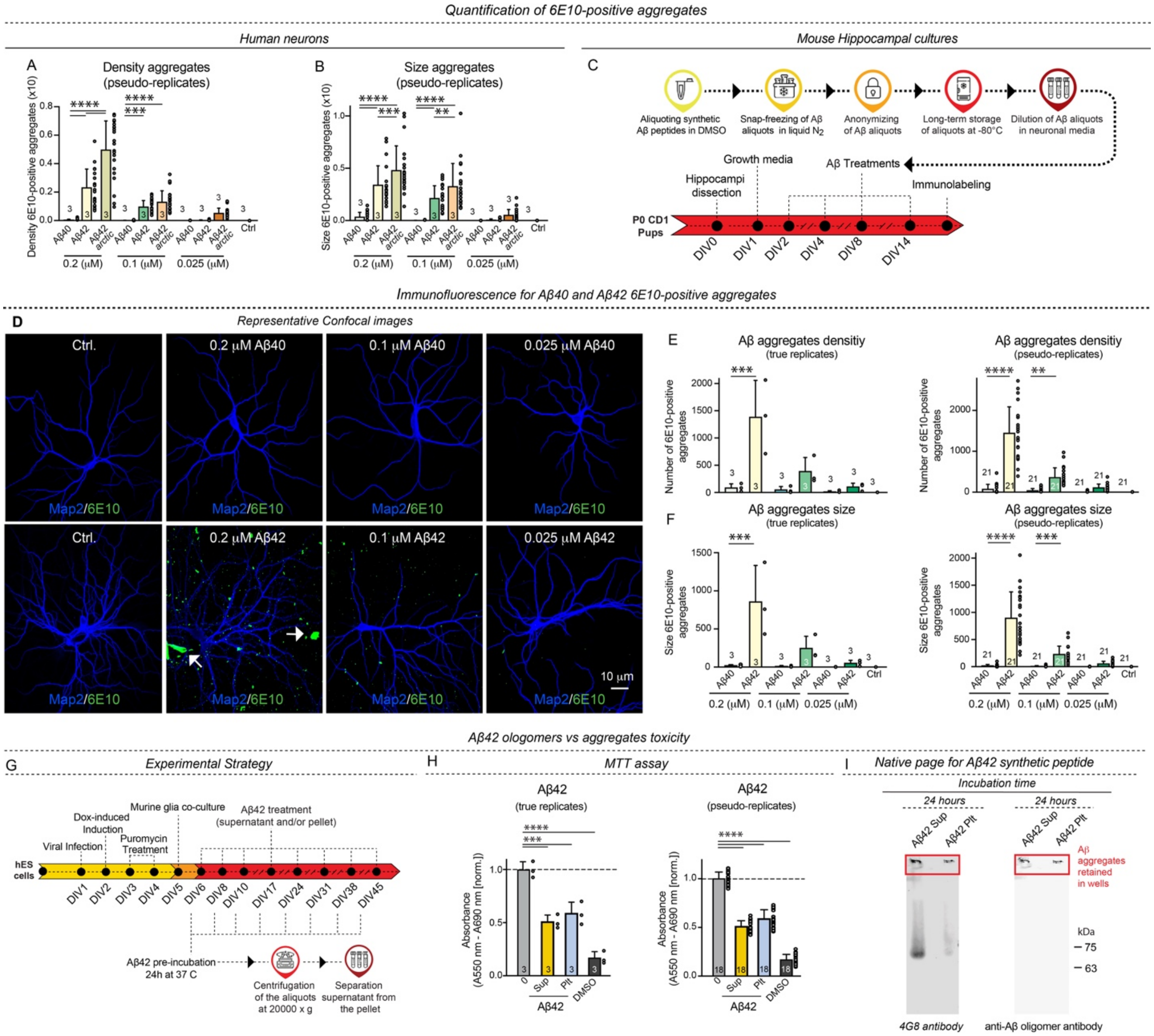

Supplementary Figure S2 | **6E10 immunofluorescence measurements of Aβ aggregate density and size in human induced neuron/mouse glia cultures (A & B) and in mouse primary neuronal cultures (C-F) assessed with both true- and pseudo-replicate statistics, and analysis of the toxicity of Aβ42 aggregates using a fractionation experiment (G-I)**

A & B. 6E10-positive Aβ aggregates density and size, in human neurons, analyzed as pseudo-replicates. Data are the same as those shown in Fig. 2B & C.

C-F. Analysis of Aβ aggregates using primary mouse neuron/glia cultures (**C**, Experimental strategy; **D**, representative images of mouse primary neurons labeled with dendritic marker MAP2 (blue), and Aβ marker 6E10 (green) (arrows point to 6E10-positive aggregates); **E** & **F**, summary graphs of the 6E10-positive aggregates density (**E**) and size (**F**)). Data were analyzed as both true and pseudo-replicates.

G. Experimental strategy for the Aβ42 fractionation experiments where the supernatants (Sup) and pellets (Plt) derived by the centrifugation of pre-aggregated Aβ42 aliquots were added to neuronal culture media with the same regimen as described for Figure 1A.

H. MTT assay of the supernatant and pellet fractions derived from the Aβ42 fractionation experiments. Results are reported both as true and pseudo-replicates.

I. Native PAGE followed by immunoblotting with the indicated antibodies of the supernatant and pellet fractions obtained from the Aβ42 fractionation experiments. Note that only the supernatant contains non-aggregated Aβ42 whereas both the supernatant and the pellet contain aggregated Aβ42, indicating that the toxicity demonstrated in panel H is due to aggregated Aβ42.

Human neurons used for the analyses reported in A and B were analyzed at DIV25; mouse primary neurons used for panels C-F were analyzed at DIV16; and human neurons used for the Aβ42 fractionation experiments were analyzed at DIV45. All numerical data are means ± SEM; numbers of experiments are reported in the bars (n=3); numbers of pseudo-replicates (n=18-21), intended as number of different non-overlapping fields of view analyzed for each condition, are reported in the bars. Statistical significance was assessed by one-way ANOVA with post-hoc corrections and comparing the mean of each group with control (Ctrl), with **$p < 0.01$, ***$p < 0.001$, and ****$p < 0.0001$. Nonsignificant comparisons are not indicated.

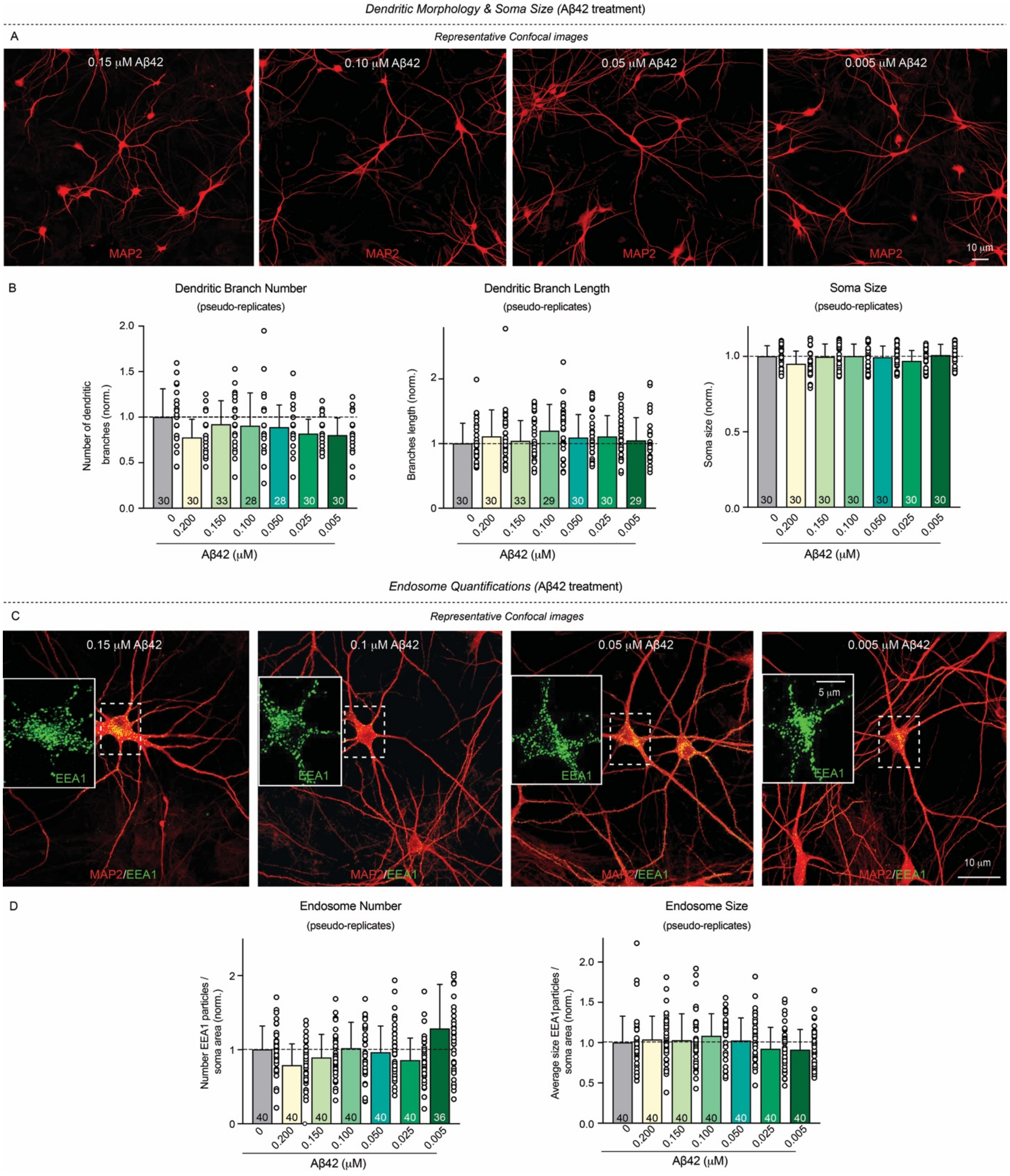

Supplementary Figure S3 | **Additional representative images from the analyses of the effect of the Aβ42 peptide on neuronal morphology (A), neuronal endosomes (C), and statistical evaluation of these analyses using pseudo-replicates instead of real replicates for quantification of the effects of Aβ peptides on neuronal morphology (B) and on endosomes (D)**

A. Additional images of human neurons labeled with anti-MAP2 antibody showing conditions for which no representative images were included in Fig. 3A.

B. Quantification of the effect of Aβ42 synthetic peptides on dendritic morphology and soma size analyzed as pseudo-replicates. Data are the same as those shown in Fig. 3B.

C. Additional representative images of neurons stained for MAP2 and the endosomal marker EEA1 (insets, expanded single-channel EEA1 images of the neuronal soma) for which no representative images were included in Fig. 3C.

D. Summary graphs of density and sizes of the EEA1-positive somatic endosomes as a function of Aβ42 concentration, with values represented as pseudo-replicates. Data are the same as those shown in Fig. 3D

All neurons were analyzed at DIV45; all numerical data are means ± SEM; numbers of pseudo-replicates (n=29-40), intended as number of different non-overlapping field of view analyzed for each condition, are reported in the bars. Statistical significance was assessed by one-way ANOVA with post-hoc corrections and comparing the mean of each group with control (Ctrl). Nonsignificant comparisons are not indicated.

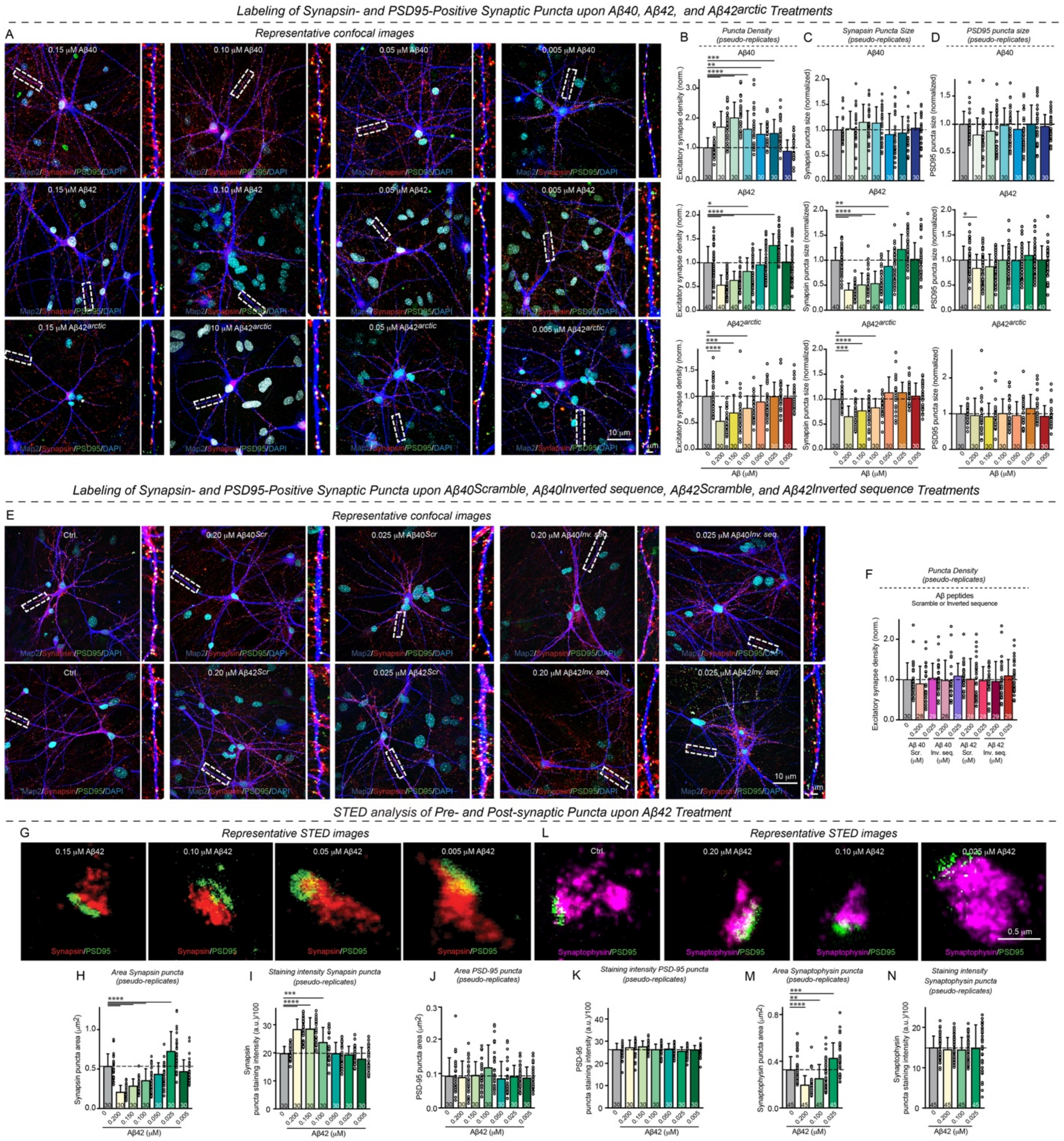

<u>Supplementary Figure S4</u> | **Additional representative images illustrating the remaining conditions that were not included in Figure 4 of the analyses of synapse density and size as a function of the treatments with Aβ40, Aβ42, their scrambled and inverted sequences versions, and Aβ42^{arctic} peptides (A-F), and further data describing the STED-imaging analyses of presynaptic and postsynaptic specializations (G-L)**

A. Additional representative images of human neurons immunolabeled for the dendritic marker MAP2 (blue), the presynaptic marker synapsin (red), and the postsynaptic marker

PSD95 (green), and additionally stained with the nuclear marker DAPI (cyan); showing conditions for which no representative images were included in Fig. 4A.

(**B**-**D**). Summary graphs of the synapse density, size of synapsin and PSD95 puncta for neurons treated with Aβ40 (**B)**, Aβ42 (**C**) or Aβ42^{arctic} (**D**). Data are reported as pseudo-replicates and are the same as those displayed in main Figure 4B, C and D.

E. Additional representative images of human neurons immunolabeled for the dendritic marker MAP2 (blue), the presynaptic marker synapsin (red), and the postsynaptic marker PSD95 (green), and additionally stained with the nuclear marker DAPI (cyan); showing conditions for which no representative images were included in Fig. 4E.

F. Summary graphs of the synapse density of synapsin- and PSD95-positive puncta for neurons treated with both scrambled and inverted sequences versions of the Aβ40 and Aβ42 peptides. Data are reported as pseudo-replicates and are the same as those displayed for true replicates in Figure 4F.

G & **L**. Additional STED images used for quantifying pre-synaptic puncta size and intensity of staining. Cells were immunostained for anti-Synapsin-1 (red), Synaptophysin (magenta) and anti-PSD95 (green).

H-I. Summary graphs, with data points reported as pseudo-replicates, of the Synapsin-positive puncta area (**H**), and its staining intensity (**I**). Data are the same as those displayed as true replicates in Figure 4H-I.

J & **K**. Summary graphs, with data points reported as pseudo-replicates, of the PSD-95-positive puncta area (**H**), and its staining intensity (**I**). Data are the same as those displayed in main Figure 4J-K.

M & **N**. Summary graphs, with data points reported both as a pseudo-replicates, of the Synaptophysin puncta area (**M**) and staining intensity (**N**). Data are the same as those displayed in Figure 4M-N.

All neurons were analyzed at DIV45; all numerical data are means ± SEM; numbers of pseudo-replicates (n=26-45), intended as number of different non-overlapping field of view analyzed for each condition, are reported in the bars. Statistical significance was assessed by one-way ANOVA with post-hoc corrections and comparing the mean of each group with control (Ctrl), with *$p < 0.05$, **$p < 0.01$, ***$p < 0.001$, and ****$p < 0.0001$. Nonsignificant comparisons are not indicated.

GCaMP6m Ca^{2+}-Imaging upon Aβ40 and Aβ42 Treatments

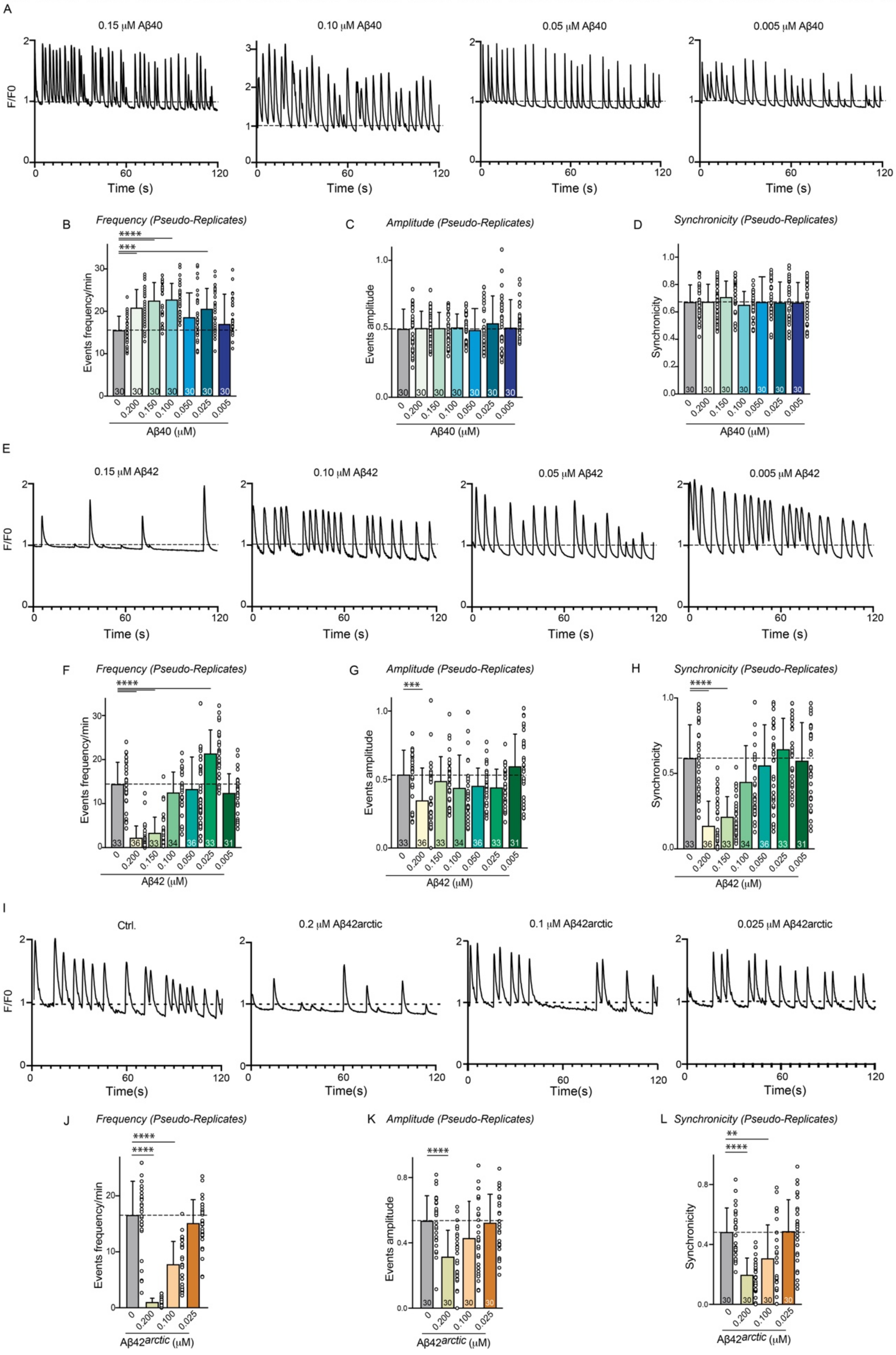

Supplementary Figure S5 | **Additional data supplementing the analyses of the effect of Aβ peptides on neuronal activity using Ca^{2+}-imaging in human neurons**

A-L. Supplementary representative Ca^{2+}-imaging traces of neurons that expressed gCamP6m and that were treated with Aβ40, Aβ42 and Aβ42^{arctic}; showing conditions for which no representative traces were included in Fig. 5 (**A, E** & **I**); and summary graphs of the indicated firing parameters of the network activity in cultured human neurons monitored as Ca^{2+}-spikes as described in Figure 5, with the results reported and statistically analyzed as pseudo-replicates(**B-D**, **F-H** & **J-L**). Data are the same as those displayed in main Figure 5B-D, F-H and J-L.

All neurons were analyzed at DIV45; all numerical data are means ± SEM; numbers of pseudo-replicates (n=30-36), intended as number of different non-overlapping field of view analyzed for each condition, are reported in the bars. Statistical significance was assessed by one-way ANOVA with post-hoc corrections and comparing the mean of each group with control (Ctrl), with $^{**}p < 0.01$, $^{***}p < 0.001$, and $^{****}p < 0.0001$. Nonsignificant comparisons are not indicated.
