# Supplementary figures and images for: Aggregation shifts amyloid-β peptides from synaptogenic to synaptotoxic
Source: J Clin Invest. 2025 Sep 30;135(24):e193407. doi: 10.1172/JCI193407 (PMC12700558; doi:10.1172/JCI193407)

**Figure 2D**

Uncropped/unedited blots

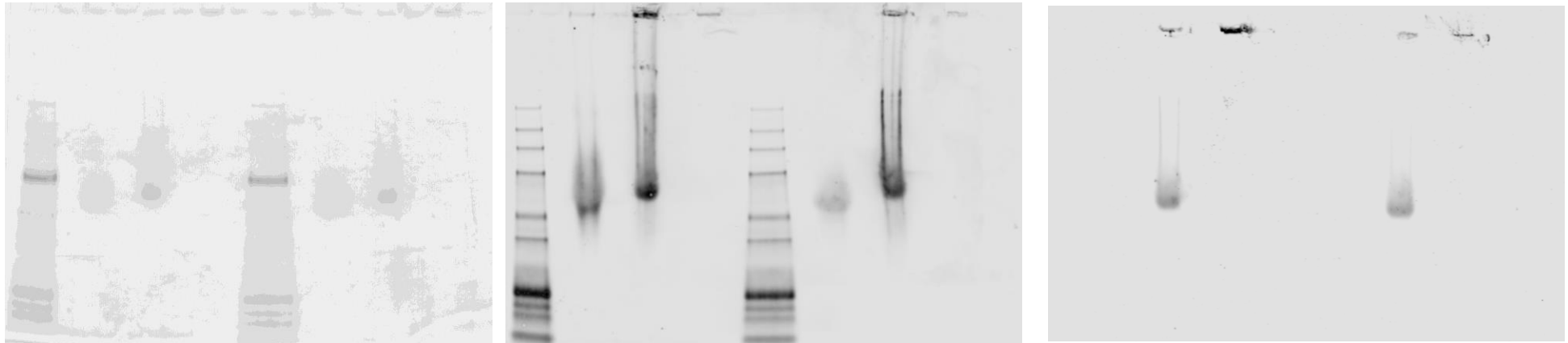

## Supplementary Figure S2I

Uncropped/unedited blots

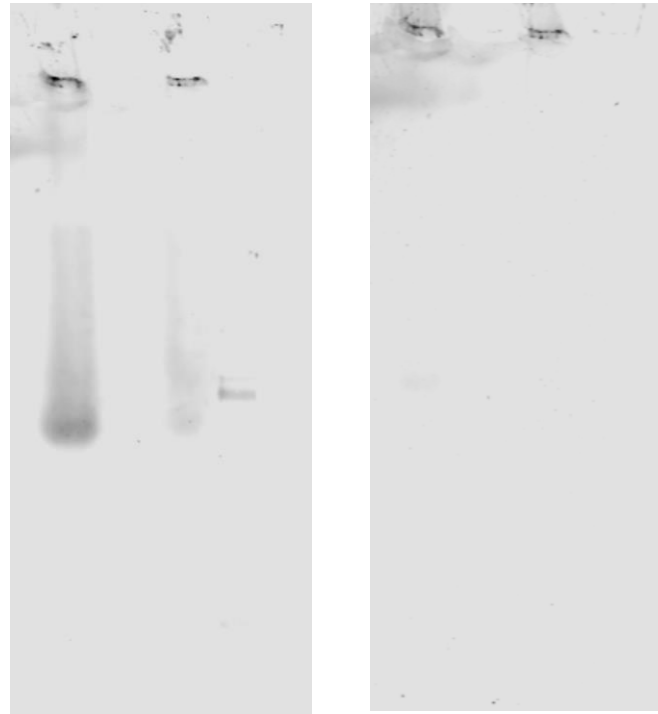

Supplement: Unedited blot and gel images [file jci-135-193407-s230.pdf]
